# Microbial and biogeochemical responses to projected future nitrate enrichment in the California upwelling system

**DOI:** 10.3389/fmicb.2014.00632

**Published:** 2014-11-20

**Authors:** Katherine R. M. Mackey, Chia-Te Chien, Adina Paytan

**Affiliations:** ^1^Earth System Science, University of California IrvineIrvine, CA, USA; ^2^Earth and Planetary Sciences, University of California Santa CruzSanta Cruz, CA, USA; ^3^Institute for Marine Science, University of California Santa CruzSanta Cruz, CA, USA

**Keywords:** global change, phytoplankton, upwelling, nutrient cycling, Iron limitation, nitrogen limitation, diatom

## Abstract

Coastal California is a dynamic upwelling region where nitrogen (N) and iron (Fe) can both limit productivity and influence biogeochemistry over different spatial and temporal scales. With global change, the flux of nitrate from upwelling is expected to increase over the next century, potentially driving additional oceanic regions toward Fe limitation. In this study we explored the effect of changes in Fe/N ratio on native phytoplankton from five currently Fe-replete sites near the major California upwelling centers at Bodega Bay and Monterey Bay using nutrient addition incubation experiments. Despite the high nitrate levels (13–30 μ M) in the upwelled water, phytoplankton at three of the five sites showed increased growth when 10 μ M nitrate was added. None of the sites showed enhanced growth following addition of 10 nM Fe. Nitrate additions favored slow sinking single-celled diatoms over faster sinking chain-forming diatoms, suggesting that future increases in nitrate flux could affect carbon and silicate export and alter grazer populations. In particular, solitary cells of *Cylindrotheca* were more abundant than the toxin-producing genus *Pseudonitzschia* following nitrate addition. These responses suggest the biogeochemistry of coastal California could change in response to future increases in nitrate, and multiple stressors like ocean acidification and hypoxia may further result in ecosystem shifts.

## Introduction

Coastal upwelling regions along eastern boundary currents are the most productive marine ecosystems, supporting complex ecological networks and economically important fisheries. These systems experience a high degree of natural spatial and temporal variability with respect to biological, chemical, and physical characteristics. Upwelling is typically a seasonal phenomenon, where alongshore winds drive sub-surface, nutrient rich waters toward the sunlit surface layers, enriching them with the macronutrients nitrogen (N) and phosphorus (P) (Pennington and Chavez, 2000; Chavez and Messie, 2009). Other factors, such as the width and depth of the continental shelf at the upwelling site and internal cycling of elements play a role in determining the flux of trace metals to surface waters (Bruland et al., 2001; Biller and Bruland, 2013; Biller et al., 2013).

Coastal upwelling regions face threats from anthropogenic global change, including changes in stratification patterns due to sea surface temperature (SST) warming, altered nutrient chemistry, increased hypoxia, and ocean acidification (as reviewed in Capone and Hutchins, 2013). For example, decreased ventilation of the Pacific Ocean due to increased stratification in the gyres is expected to alter seawater chemistry, increasing the nitrate inventory and decreasing the oxygen content in waters that are upwelled (Rykaczewski and Dunne, 2010). As a result, the flux of nitrate in the coastal California upwelling system is expected to be 64% greater in the year 2100 compared to preindustrial times (or 28% over modern day values; Rykaczewski and Dunne, 2010).

**Figure 1:**
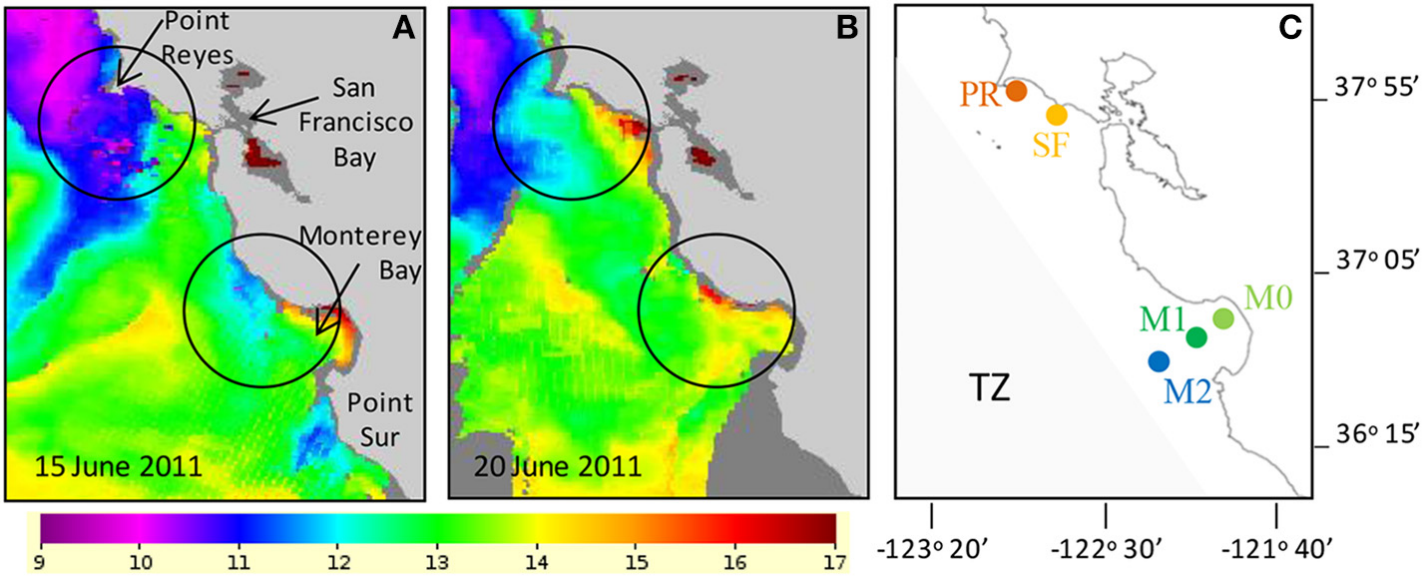
**Sea surface temperature indicated that colder upwelled water was present on June 15 (A) and that upwelling had relaxed and surface water began to warm by June 20 (B)**. Temperature scale is in °C. Areas of intense upwelling in this study are circled on each map. **(C)** Map showing the locations where incubation water was collected (see methods for latitude and longitude). Shaded region in **(C)** indicates the approximate location of the “transition zone” (TZ) described by Biller and Bruland (2014).

Iron (Fe) availability governs nitrate drawdown in many coastal upwelling systems and is strongly influenced by the physical and bathymetric characteristics of each site. Along the California coast, biomass at locations with narrow continental shelves can become Fe-limited due to the low suspended sediment levels and high nitrate concentrations from upwelling (Hutchins and Bruland, 1998; Firme et al., 2003). Recent work by Biller and Bruland (2014) expanded these regions to include the coastal California transition zone (TZ, Figure 1C), which is an offshore region with high nitrate from upwelled waters advected offshore. As the water moves offshore, labile Fe is consumed leading to Fe limitation and excess nitrate. These Fe-limited regions typically have iron-to-nitrogen (Fe/N) ratios below 0.2 nmol Fe/1 μ mol N, and have been designated as high nutrient low chlorophyll (HNLC) regions due to the relatively high residual nitrate and lower than expected chlorophyll levels. Other studies in the region have shown that despite high nitrate concentrations in the water, phytoplankton biomass remains nitrogen-limited (Kudela and Dugdale, 2000; Mackey et al., 2010), with other nutrients like phosphorus influencing physiology and competition between taxonomic groups (Nicholson et al., 2006; Mackey et al., 2012).

Iron availability in coastal California and other HNLC regions is also known to influence phytoplankton community structure and biogeochemistry. Large scale iron addition experiments conducted in the Southern Ocean and equatorial Pacific HNLC regions favored blooms of diatoms over other types of phytoplankton (Coale et al., 1996; Boyd et al., 2000; Smetacek et al., 2012), affected diatom speciation (Tsuda et al., 2003, 2005; Assmy et al., 2007), and altered grazing rates (Tsuda et al., 2006). Iron additions have also been shown to stimulate the toxin producing diatom *Pseudo-nitzschia* spp. in the equatorial Pacific Ocean (Silver et al., 2010; Trick et al., 2010). In Fe-limited areas of coastal California, selective enrichment of chain-forming diatoms occurred following iron addition, and diatoms formed more heavily silicified cells (Hutchins and Bruland, 1998). Iron availability therefore has the potential to influence the type of phytoplankton that dominate blooms following upwelling, affect the uptake and cycling of other nutrients, and alter the relative proportions of silica and carbon sequestration by the biological pump. In addition blooms with different types of phytoplankton (size, density, TEP production etc.) have different sinking velocities, also impacting carbon sequestration by the biological pump (De La Rocha and Passow, 2007). Larger cells or chain-forming cells have faster sinking rates and therefore sequester carbon more efficiently than smaller cells.

A number of factors sensitive to global change influence the supply and bioavailability of Fe in coastal upwelling regions. Biological ligand production, ocean acidification, hypoxia, rainfall, groundwater discharge, and deposition of anthropogenic aerosols influence Fe supply and biogeochemistry in coastal waters. The predicted increased supply of nitrate in the future (Rykaczewski and Dunne, 2010) could likewise shift Fe limitation regimes for biomass by changing the Fe/N ratio in the water. For example, a 50% increase in nitrate supply could drive certain Fe-replete California waters below the Fe/N limitation threshold (Capone and Hutchins, 2013), potentially expanding Fe limitation in the California upwelling region.

To understand how Fe-replete waters in coastal California could respond to changing nitrate fluxes (and hence changing Fe/N ratios) in the future, we conducted nutrient addition incubation experiments with water collected at five Fe replete sites from Monterey Bay to Point Reyes several days after upwelling when Fe and nitrate levels remained elevated. The goal of the study was to expand our understanding of how N and Fe availability and ratios could influence phytoplankton growth and physiology in modern-day and future coastal California waters and to assess the spatial variability in this response. These coastal sites were selected to encompass various distances from shore and represent major upwelling centers such that a range of nutrient conditions could be tested. We used phytoplankton cell counts to determine if there is a phytoplankton community shift toward faster sinking chain-forming diatoms as observed by Hutchins and Bruland (1998) and Silver et al. (2010) in Fe-limited regions following Fe fertilization. Finally, based on nutrient measurements, we show that only in some cases do Fe and N cause shifts in the drawdown ratios of N:P and Si:N. We discuss the implications of these findings to the export of C and Si.

## Materials and methods

### Remote sensing and mooring data

*In situ* wind speed, wind direction, SST and nitrate concentrations at station M1 were obtained from http://www.mbari.org/oasis/. Satellite images of SST were obtained from NOAA POES AVHRR, LAC, 0.0125°, day and night, courtesy of NOAA NWS Monterey and NOAA CoastWatch. Oceanographic and atmospheric conditions were monitored prior to the experiment to identify a period of upwelling followed by relaxation. The incubation experiments were conducted several days after upwelling occurred when upwelling-favorable winds had relaxed and SST values indicated surface waters were warming (Figures 1A,B).

### Incubation setup and sampling

Incubation experiments were conducted to determine the effect of N and Fe on phytoplankton using seawater collected from five sites as shown in Figure 1C a few days after the relaxation of upwelling. Water temperature and salinity were used to identify water masses by assuming that upwelled water warms ~0.5–1°C per day (K. Bruland, personal communication) and is more saline than surface water from the North Pacific. Water from Drakes Bay near Point Reyes (PR; 37°59′23.2″N, 122°58′52.2″W) and north of the mouth of San Francisco Bay (SF; 37°55′30.7″, 122°49′41.7″W) was collected on June 20, 2011 (Figure 1C) aboard the R/V Shana Rae. These two sites are located to the south and downstream of the major upwelling center located at Bodega Bay. Water was collected from the Monterey Bay moorings M0 (36°49.442N 121°56.967W) and M1 (36°45.190N 122°01.525W) and offshore of Monterey Bay near mooring M2 on June 22, 2011 using a small motor boat. In this study we refer to the offshore site as “M2,” although its actual location (36°42.382N 122°13.798W) differs slightly from the official location of the offshore mooring. Station M0 is located within Monterey Bay. Station M1 is situated directly downstream of the Monterey Bay upwelling center.

Surface (5 m) seawater was collected at each site into trace metal clean, seawater rinsed carboys. Water was transported in the dark back to Long Marine Laboratory in Santa Cruz, CA where the experiments were conducted. The following protocol was followed while setting up the experiments for each site. Three baseline samples to characterize the collected seawater were immediately collected and processed for each of the measurements described below. Water was then dispensed into acid cleaned, sample rinsed, transparent polycarbonate bottles (500 mL per bottle, 9 bottles per treatment). All materials used in the experiment were rendered trace metal clean prior to use. Plastic ware (including incubation bottles, carboys, and sample bottles) was soaked overnight in heated, ultrapure 10% hydrochloroic acid (HCl; Optima), rinsed several times with MilliQ water, and stored individually inside clean plastic bags prior to use. During sampling, bottles were opened within a laminar flow hood to minimize contamination from airborne particles.

The Fe/N ratio of the seawater was manipulated by making additions of Fe or nitrate. Different treatments used in the experiments included control (no addition), 10 μ M sodium nitrate (hereafter “nitrate”), or 10 nM iron prepared from atomic absorption standard stock in HCl (Sigma). The nitrate addition was intended to mimic the increase in nitrate projected for the California upwelling system in the year 2100 (Rykaczewski and Dunne, 2010), and the Fe was intended to at least double ambient concentrations of Fe in this area (Bruland et al., 2001). Following the addition of nutrient spikes (time zero), three bottles were immediately sampled for each of the measurements described below. The remaining bottles (6 per treatment) were placed in a large pool through which ocean water was continually circulated. A neutral density shade cloth was placed over the pool to decrease the irradiance by half, and the shading does not alter the spectral quality of the light. Three bottles from each treatment were collected 48 and 96 h after time zero and were processed as described below.

Bottle incubation experiments are useful for examining short term changes in phytoplankton community composition and physiology, particularly with respect to changing water chemistry including nutrients. The impact of grazers is more difficult to assess from bottle experiments, particularly when the incubation volume is low. This is particularly true for large grazers, like copepods, that are far less abundant than phytoplankton and may not survive in the bottles over time. Bottle experiments also have difficulty capturing variability in light due to mixing of surface water. These factors must be taken into consideration when interpreting bottle experiment results, where grazing and other factors do not perfectly mimic the environment.

### Water chemistry

Nutrient samples were 0.45 μm filtered and frozen until analysis. Nutrient analyses for nitrate (with nitrite), soluble reactive phosphorus (SRP), and silicate were carried out on a Lachat Autoanalyzer (FIA, Lachat Instruments Model QuickChem 8000). Total dissolved Fe was measured in the seawater from each site. Samples were 0.2 μm filtered in a laminar flow hood and acidified to pH <2.0 with concentrated trace metal grade nitric acid (final concentration 0.02 M) at least 48 h before analysis. The pH was adjusted to 6 with ammonium acetate and ammonium hydroxide, and samples were concentrated using Nobias Chelate-PA1 resin (HITACHI High Technologies, Japan) to remove the seawater matrix (Sohrin et al., 2008; Biller and Bruland, 2012). The Fe was eluted with 1 M trace metal grade nitric acid and analyzed for by HR-ICPMS (Thermo Element XR). The detection limit for Fe was 0.91 nM.

### Phytoplankton growth

Phytoplankton growth was assessed based on measurements of chlorophyll *a* (chl *a*) and direct enumeration of cells using flow cytometry and microscope cell counts. For chl *a* measurement, 50 mL of incubation water was filtered through GFF filters (Whatman) and the filters were flash frozen and stored at −80°C until analysis. Filters were extracted in 5 mL 90% acetone (Optima) in the dark at −20°C for 24 h. Fluorescence was measured on a Turner Designs AU10 and converted to concentration via a standard curve calibration.

Flow cytometry samples were collected and fixed with a final concentration of 0.1% glutaraldehyde and flash frozen and stored at −80°C until analysis. Samples were thawed on ice and run on an Influx flow cytometer (Becton Dickinson). Using FlowJo software, phytoplankton cells were identified from their red (692 nm) chlorophyll autofluorescence signal. *Synechococcus* cells were identified by their phycoerythrin signal (572 nm).

Samples for microscope analysis were fixed with a final concentration of 2% formalin and stored in the dark at 4°C in glass bottles. A settling chamber was used to concentrate 2–10 mL of sample, and cells were viewed on an inverted microscope. Typically ~1000 cells were counted per sample, but never less than 300, and replicate samples were randomly included to check for accuracy. Cells >4 μm in diameter were identified and enumerated as pennate diatoms, centric diatoms, or “others” (Supplemental Figures 1–8). Pennate and centric diatoms were further grouped according to whether they occurred as single cells or in chains of two or more cells. In the text their relative abundances are categorized as rare (<1%), present (1–9%), common (10–49%) or abundant (>50%). Statistical significance was determined using ANOVA followed by pairwise comparisons using a Bonferroni correction. Cell counts in the Fe and N treatments at each site were compared to the control (two comparisons per site).

## Results

### Remote monitoring

**Figure 2:**
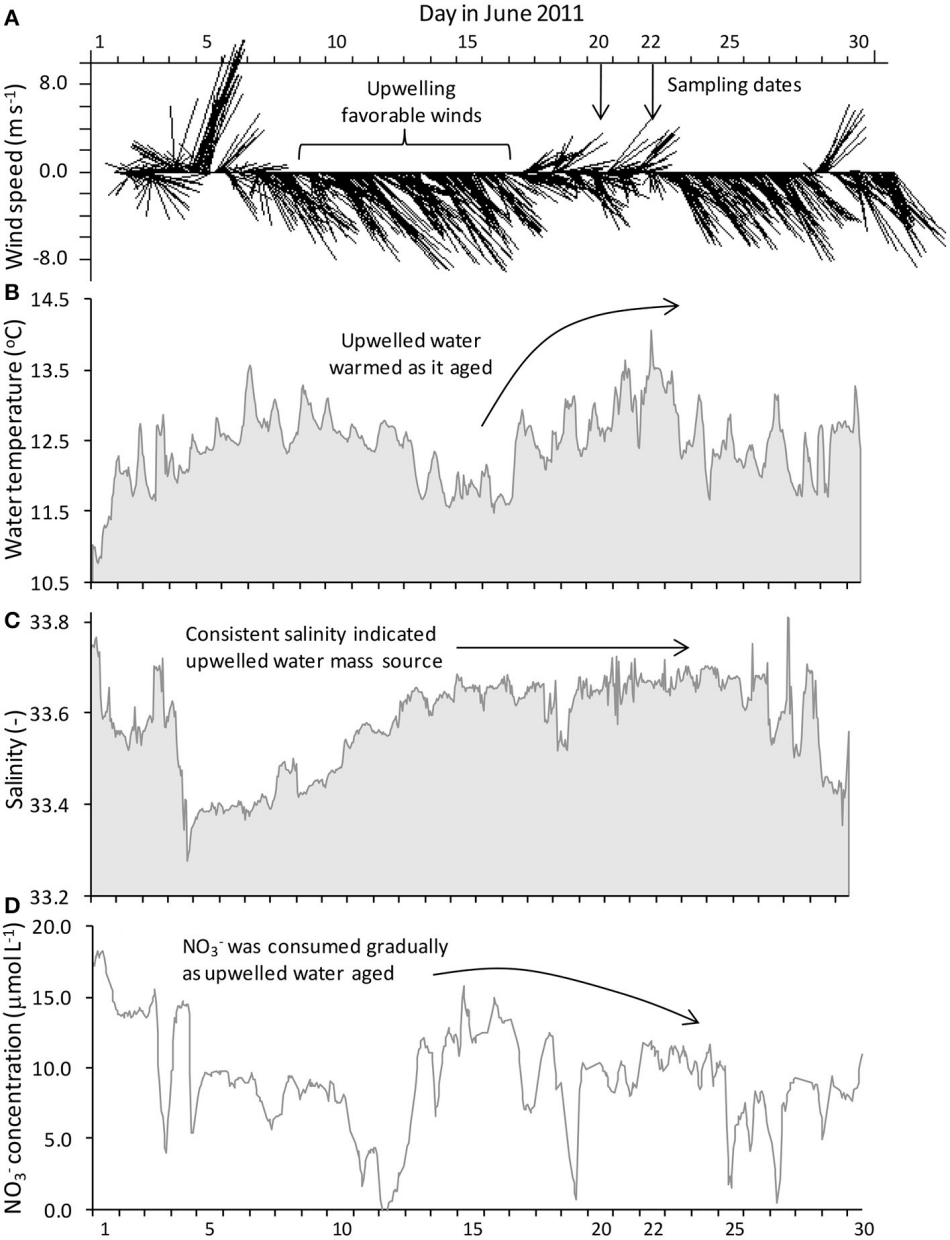
***In situ* mooring data for station M1 showing (A) hourly gridded wind velocity, (B) sea surface temperature, (C) salinity, and (D) nitrate concentration**. Upwelling favorable winds occurred in the days prior to sampling and were accompanied by cold, saline water with high nitrate levels. Winds relaxed beginning June 17, causing upwelling to cease and allowing surface waters to warm as nitrate was drawn down. Incubation water was collected on June 20 and 22.

Wind velocity recorded at the M1 mooring indicated that upwelling favorable winds reached speeds up to ~8 m s^−1^ during the period of June 10–16. The winds consistently blew from the northwest to the southeast during this period (Figure 2A). Cold water masses were observed from the AVHRR satellite during these dates along the California coast, indicating upwelling of deep water was occurring. By June 15, a large, cold (9–10°C) upwelled water mass was located surrounding Point Reyes and was centered offshore of Bodega Bay, and smaller upwelled water masses were located north of Monterey Bay and at Point Sur (Figure 1A). Upwelling favorable winds relaxed from June 17 to 23, and the wind direction became more variable (Figure 2A). By June 20 the amount of cold water being upwelled had declined and was more restricted to the coastline north of Point Reyes (Figure 1B). The water masses south of Point Reyes and near Monterey Bay continued to warm once upwelling ceased (Figure 1B).

Temperature, salinity, and nitrate data from the mooring at M1confirmed the pattern of upwelling and relaxation observed from the satellite images (Figure 2, Supplemental Figure 9). At M1, surface waters became progressively colder and more saline from June 10 to 16 while upwelling favorable winds prevailed (Figures 2B,C, Supplemental Figure 9). By June 15 a cold (~11.5°C), saline (~33.6) water mass was present at the mooring. Upon relaxation of upwelling favorable winds (June 17–23), the water mass began to warm. Relatively stable salinity indicated that the warmer temperatures were associated with the same upwelled water mass rather than from intrusion of waters from the less saline California current.

The nitrate concentration at mooring M1 reached its highest level (~15 μmol L^−1^; Figure 2D) in surface waters by the end of the upwelling period (June 15–16). Nitrate declined gradually by ~5 μmol L^−1^ from June 17 to 23 once upwelling favorable winds relaxed; however, nitrate levels were still relatively high (~10 μmol L^−1^) and had not been consumed entirely by June 23.

**Figure 3:**
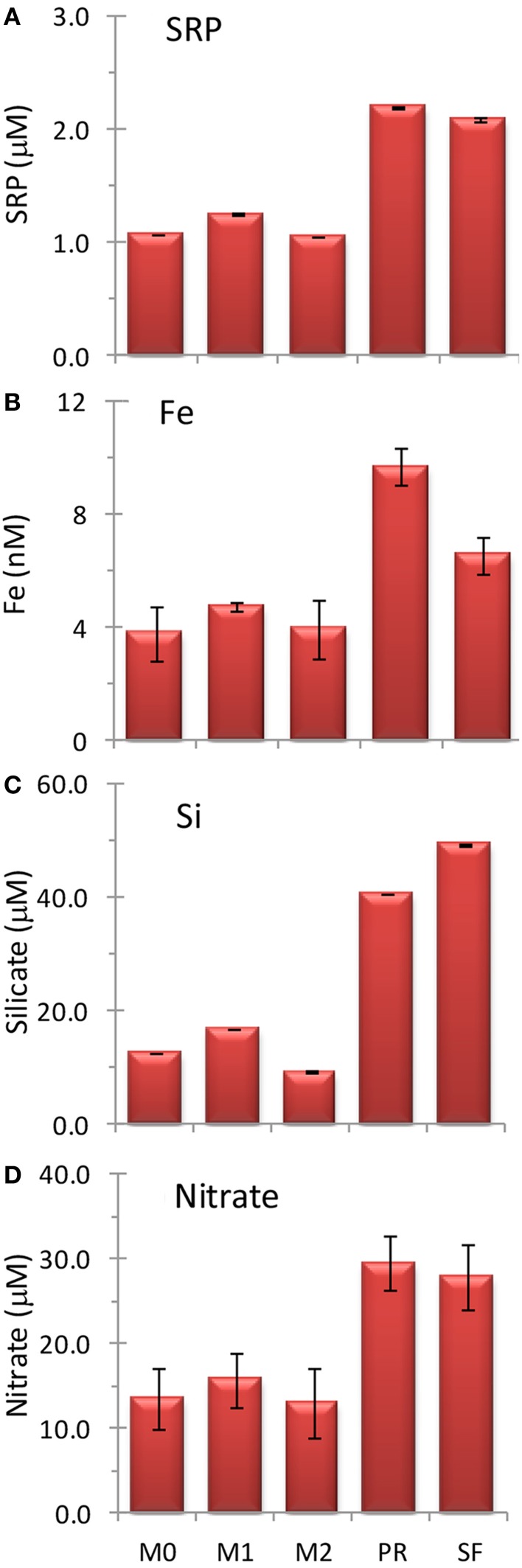
**Initial concentrations of (A) soluble reactive phosphorus, SRP, (B) Fe, (C) silicate, and (D) nitrate at the five sampling locations**. Error bars show standard deviation.

**Figure 4:**
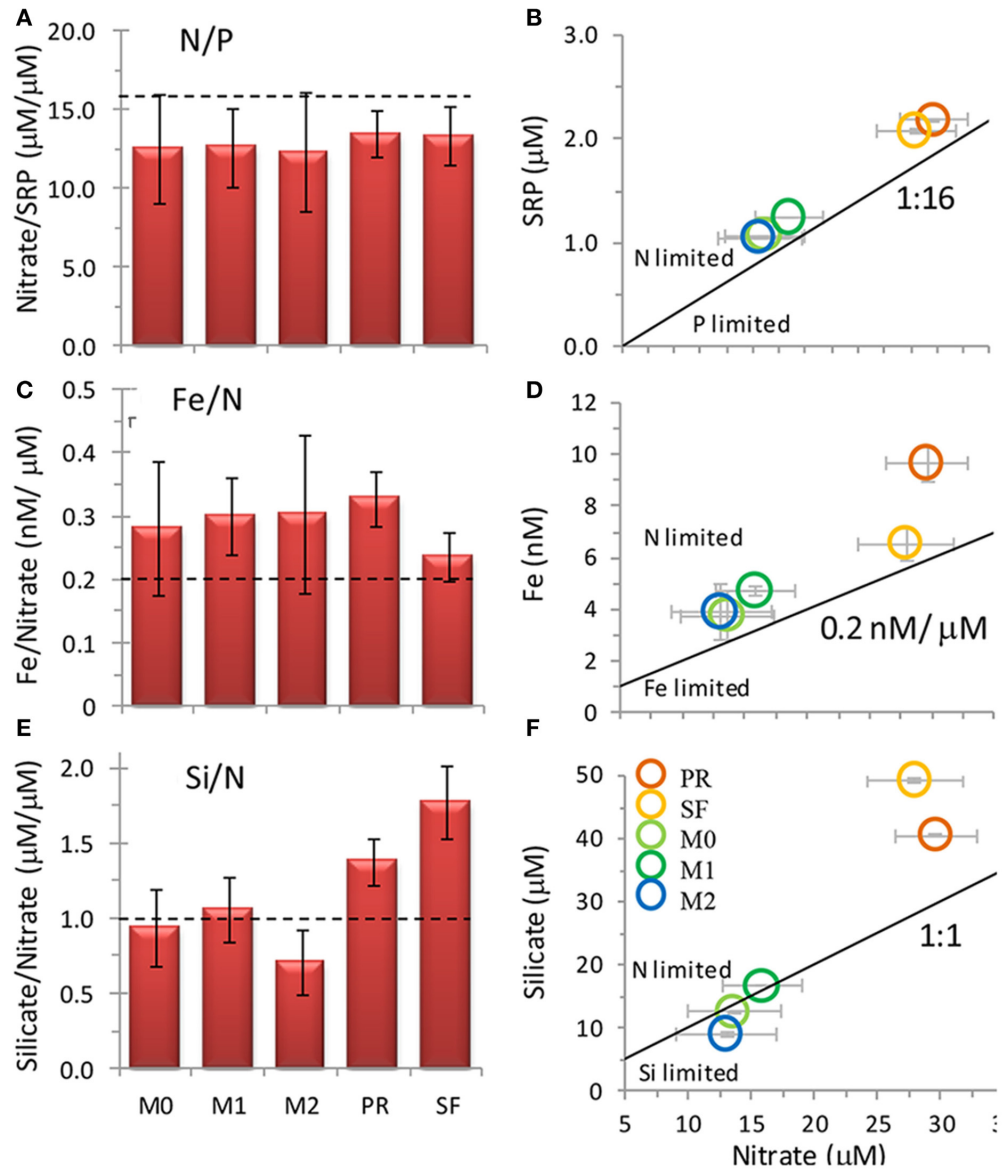
**Nutrient ratios of (A,B) SRP: nitrate, (C,D) Fe: nitrate, and (E,F) silicate: nitrate at the five sampling locations when the incubation water was collected**. Legend for (**B,D,F** shown in **F**). Error bars show standard deviation. Broken lines in **(A,C,E)** and solid lines in **(B,D,E)** show typical ratios of diatom cellular nutrient quotas.

Water was collected for the incubation experiments once upwelling favorable winds had relaxed for several days and surface water had warmed 1–2°C based on remote monitoring data as described above (e.g., June 20 for stations PR and SF, and June 22 for stations M0, M1, and M2). Nutrient and Fe levels were lower at stations M0, M1, and M2 than at stations PR or SF (Figures 3A–D). The ratio of N/P was similar among all five sites (Figures 4A,B), whereas the ratio of Si/N was higher at stations PR and SF than at M0, M1, or M2 (Figures 4E,F). The Fe/N ratio was not statistically different among the five sites at *p* < 0.05 (Figures 4C,D). The incubation results for each of the stations are discussed below.

### Incubation experiments

#### Drakes Bay at Point Reyes (station PR)

**Figure 5:**
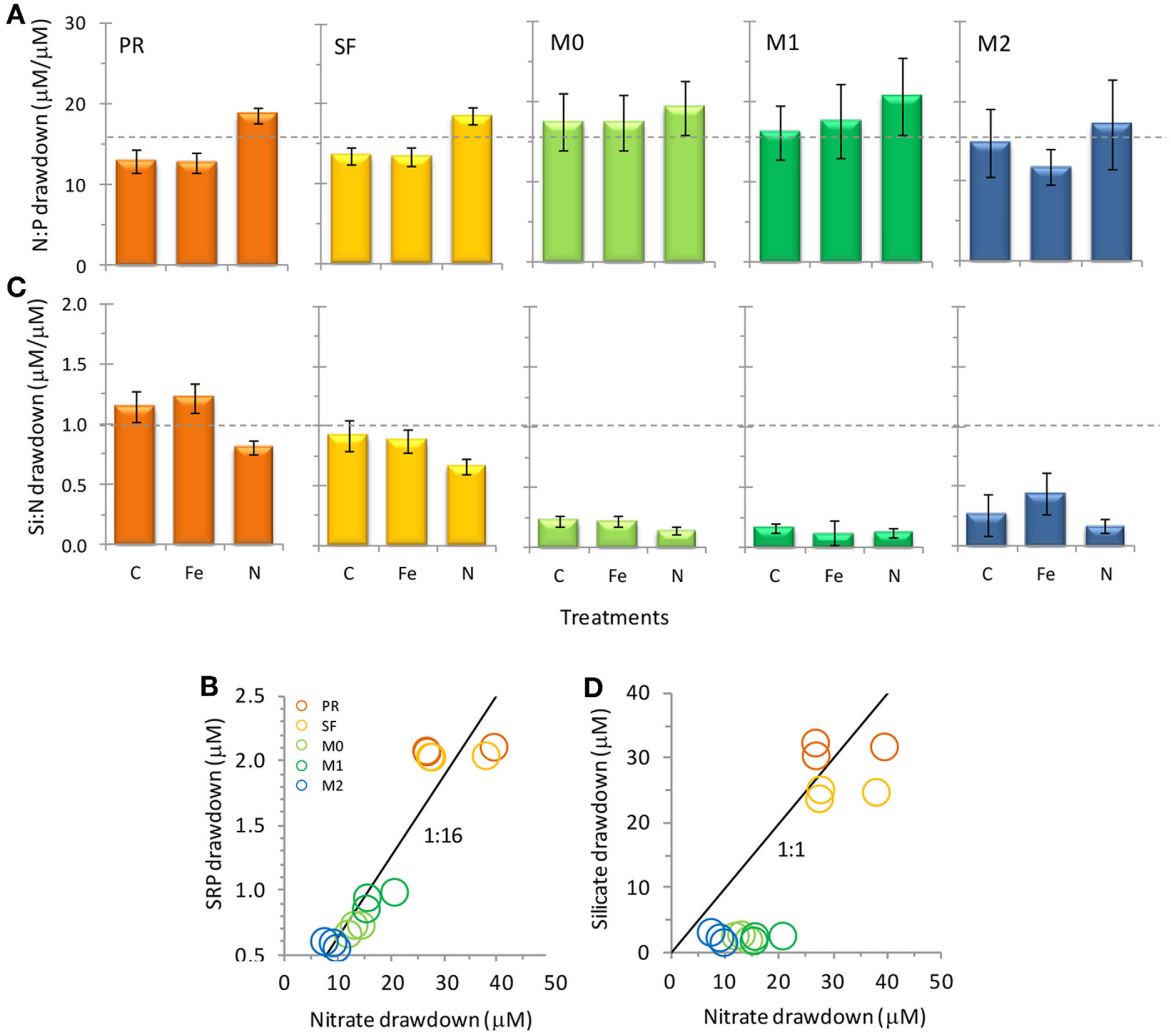
**Nutrient drawdown ratios of (A,B) N/P and (C,D) Si/N for the five stations in this study**. For **(B,D)** values are shown for all three treatments for each station. Values are calculated as the ratios of final minus initial nutrient concentrations. Error bars show standard error. Broken lines in **(A,C)** and solid lines in **(B,D)** show typical ratios of diatom cellular nutrient quotas.

Initial nutrient concentrations at station PR were 29.5 ± 3.3 μmol L^−1^ nitrate, 2.2 ± 0.0 μmol L^−1^ SRP, 40.8 ± 0.1 μmol L^−1^ silicate, and 9.2 ± 0.65 nmol L^−1^ Fe, with a N/P ratio of 13.4 ± 1.5, a Si/N ratio of 1.4 ± 0.1, and a Fe/N ratio of 0.33 ± 0.0 nM/μ M (Figures 3, 4). The range of N/P drawdown ratios for all treatments was 13–19, and the range for Si/N was 0.8–1.2 (Figure 5).

**Figure 6:**
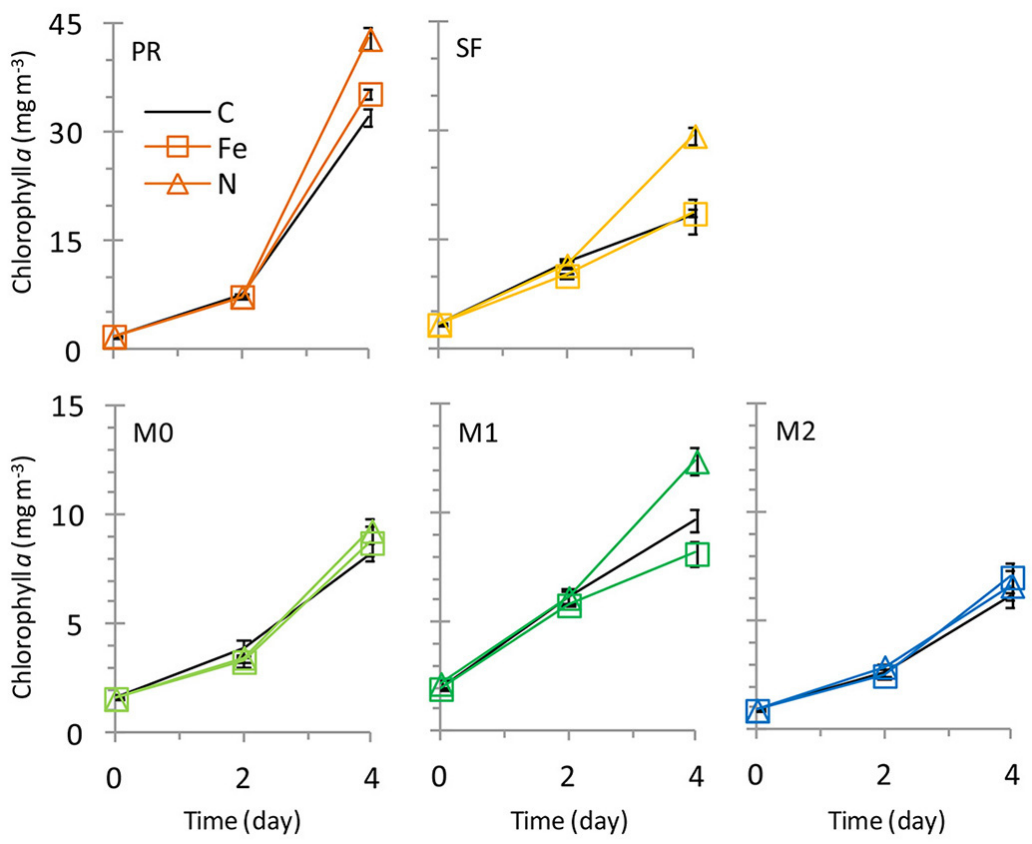
**Growth curves showing chlorophyll *a* over time**. Nitrogen additions increased growth at stations PR, SF, and M1. Error Bars show standard error.

**Figure 7:**
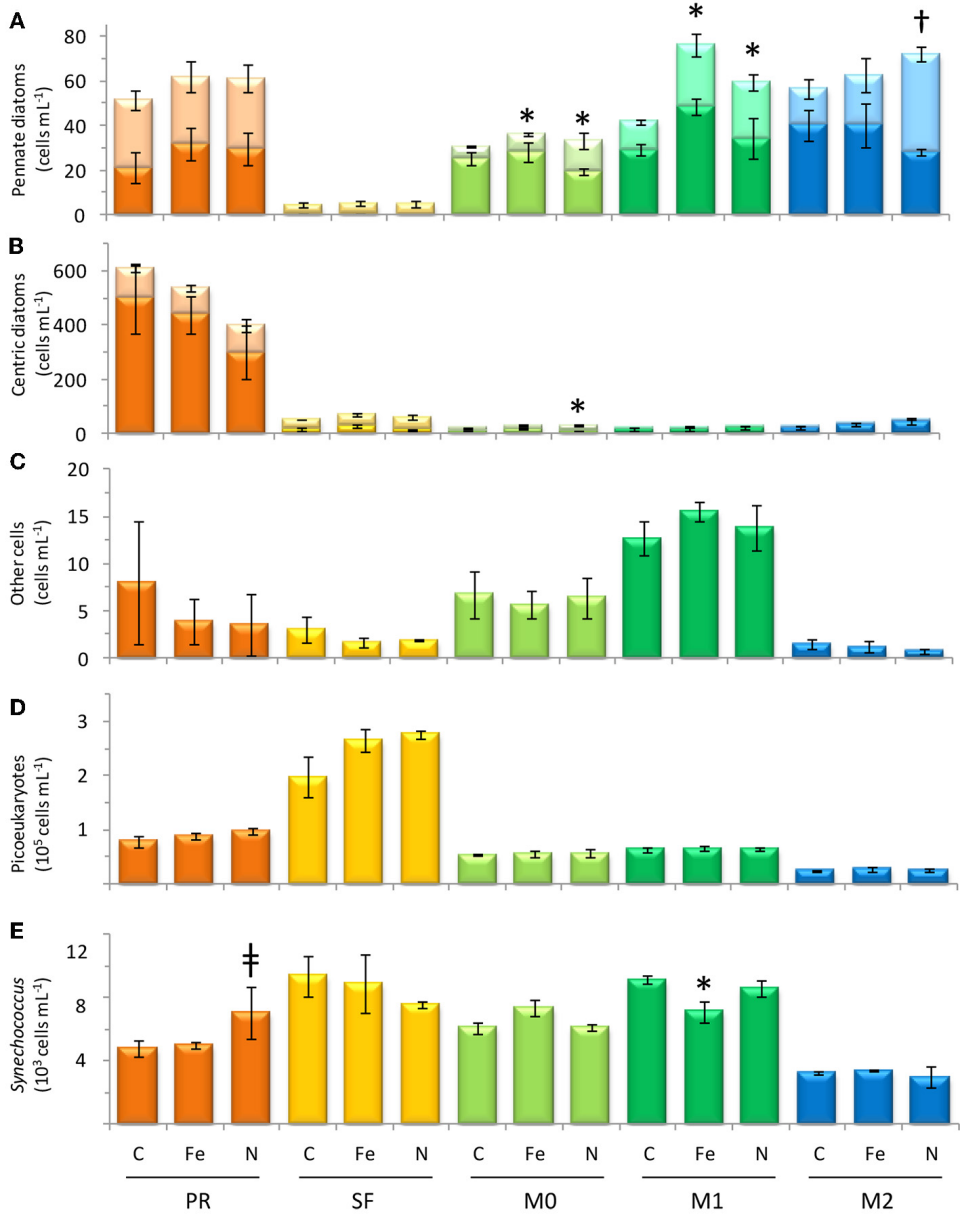
**Cell count data from the final time point of each incubation, showing concentrations of (A) pennate diatoms, (B) centric diatoms, (C) dinoflagellates, and other cells >4μm, (D) picoeukaryotes, and (E) *Synechococcus***. Error bars show standard error. Light-colored stacked bars in **(A,B)** show single celled diatoms and dark-colored stacked bars show chain-forming cells. Symbol † denotes *p* < 0.05; symbol ^*^denotes *p* < 0.10; symbol ‡ denotes *p* < 0.15.

**Figure 8:**
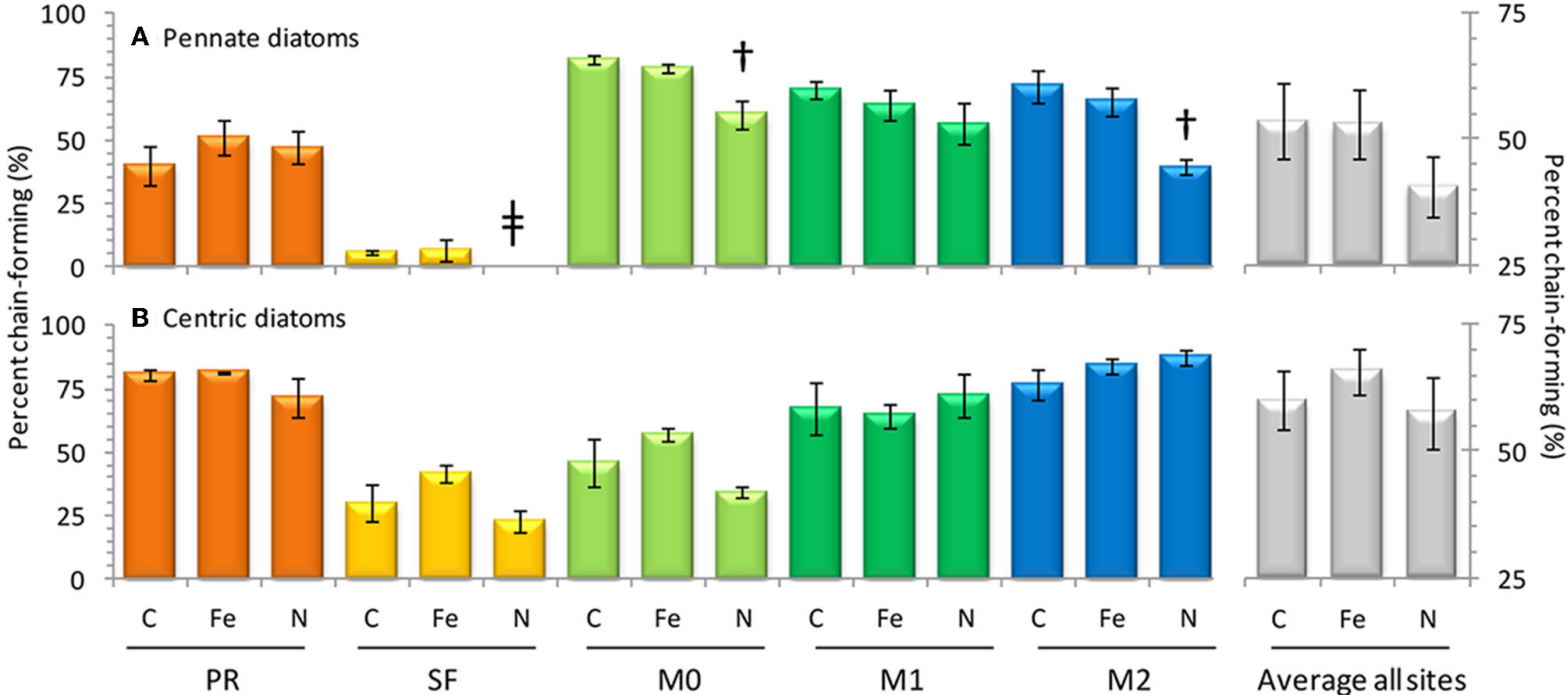
**Observations of chain-forming behavior for pennate diatoms (above) and centric diatoms (below)**. At right data from all sites has been (gray bars, *n* = 15 for each treatment). Error bars show standard error. Symbol † denotes *p* < 0.05; symbol ^*^denotes *p* < 0.10; symbol ‡ denotes *p* < 0.15.

The initial chl *a* concentration was 1.9 ± 0.1 mg m^−3^, and the range of final concentrations for all treatments was 32.2–43.0 mg m^−3^. The N treatment resulted in a higher chl *a* increase than other treatments (Figure 6). The phytoplankton population at Point Reyes was dominated by several species of *Thalassiosira*, and the majority of these were chain-forming in all treatments by the end of the incubation. Pennate diatoms were present at this site across all treatments and between 40 and 57% of them were chain-forming (Figures 7, 8; cell concentration data is given in Supplemental Table 1). Among the picophytoplankton, picoeukaryotes (range across treatments = 78–104 × 10^3^ cells mL^−1^) outnumbered *Synechococcus* (range across treatments = 5–7 × 10^3^ cells mL^−1^) (Figure 7).

#### Coastline north of San Francisco Bay (Station SF)

Initial nutrient concentrations at station SF were 27.9 ± 3.8 μmol L^−1^ nitrate, 2.1 ± 0.0 μmol L^−1^ SRP, 49.5 ± 0.2 μmol L^−1^ silicate, and 6.2 ± 0.61 nmol L^−1^ Fe with a N/P ratio of 13.3 ± 1.8, a Si/N ratio of 1.8 ± 0.2, and a Fe/N ratio of 0.24 ± 0.04 nM/μ M (Figures 3, 4). The range of N/P drawdown ratios for all treatments was 13–18, and the range for Si/N was 0.7–1.0 (Figure 5).

The highest initial chl *a* concentration (3.3 ± 0.0 mg m^−3^) was observed at this site. By the end of the experiment chl *a* levels in the Fe treatments clustered with the control (16.0-18.4 mg m^−3^), and N caused an increase in chl *a* (29.4 ± 1.1 mg m^−3^) above the control (Figure 6). The highest picophytoplankton abundances were observed at this site, where picoeukaryote abundances ranged from 198 to 276 × 10^3^ cells mL^−1^, and *Synechococcus* abundances ranged from 6 to 9 × 10^3^ cells mL^−1^ (Figure 7). Among phytoplankton larger than 4 μm, small centric diatoms were abundant and the majority existed as single cells (Figures 7, 8). Some of these small centric cells fell below the 4 μm size category and are instead included in the picoeukaryote category (enumerated by flow cytometry). Treatment with Fe caused an increase in chain-forming centric diatoms relative to the control (Figures 7, 8). *Coscinodiscus* was also observed at this site, though rare. Pennate diatoms were observed, and chain-forming pennate cells were very rare (<<1%). The majority were *Cylindrotheca* spp.

#### Inner Monterey Bay (Station M0)

Initial nutrient concentrations at station M0 were 13.5 ± 3.7 μmol L^−1^ nitrate, 1.1 ± 0.0 μmol L^−1^ SRP, 12.7 ± 0.0 μmol L^−1^ silicate, and 3.6 ± 0.93 nmol L^−1^ Fe, with a N/P ratio of 12.5 ± 3.4, a Si/N ratio of 0.94 ± 0.3, and a Fe/N ratio of 0.28 ± 0.11 nM/μ M (Figure 4). The range of N/P drawdown ratios for all treatments was 17–19, and the range for Si/N was 0.1–0.2 (Figure 5).

The initial chl *a* concentration was 1.6 ± 0.1 mg m^−3^, and all treatments had similar final concentrations as the control (8.2–9.3 mg m^−3^; Figure 6). The phytoplankton population at M0 was dominated by *Pseudonitzschia* spp., and the majority of cells were chain-forming in all treatments. Bottles receiving N additions had a lower percentage (60%) of chain-forming pennate diatoms compared to the control and Fe addition treatments (78–82%, Figure 7). Centric diatoms were common at this site across all treatments, and between 34 and 57% were chain-forming (Figures 7, 8). Picoeukaryotes (range across treatments = 49–57 × 10^3^ cells mL^−1^) outnumbered *Synechococcus* (range across treatments = 6–7 × 10^3^ cells mL^−1^) (Figure 7).

#### Outer Monterey Bay (Station M1)

Initial nutrient concentrations at station M1 were 15.8 ± 3.2 μmol L^−1^ nitrate, 1.3 ± 0.0 μmol L^−1^ SRP, 16.8 ± 0.0 μmol L^−1^ silicate, and 4.5 ± 0.17 nmol L^−1^ Fe, with a N/P ratio of 12.6 ± 2.5, a Si/N ratio of 1.1 ± 0.2, and a Fe/N ratio of 0.30 ± 0.06 nM/μ M (Figures 3, 4). The range of N/P drawdown ratios for all treatments was 16–21, and the range for Si/N was 0.1–0.2 (Figure 5).

The initial chl *a* concentration was 1.9 ± 0.0 mg m^−3^, and the range of final concentrations for all treatments was 8.2–12.4 mg m^−3^. Nitrogen additions caused the greatest increase in chl *a* (12.4 mg m^−3^, Figure 6). The phytoplankton population at M1 was dominated by *Pseudonitzschia* spp., and the majority of cells were chain-forming. Bottles receiving N additions had a lower percentage (56%) of chain-forming pennate diatoms compared to the control and Fe addition treatments (64–70%, Figure 7). Centric diatoms were common at this site across all treatments, and between 64 and 72% were chain-forming (Figures 7, 8). Picoeukaryotes (range across treatments = 61–65 × 10^3^ cells mL^−1^) outnumbered *Synechococcus* (range across treatments = 6–9 × 10^3^ cells mL^−1^) (Figure 7). Treatment with Fe additions caused a decline in *Synechococcus* abundance relative to the control, whereas picoeukaryote abundances were not sensitive to any of the treatments (Figure 7).

#### Offshore of Monterey Bay (Station M2)

Initial nutrient concentrations at station M2 were 13.0 ± 4.0 μmol L^−1^ nitrate, 1.1 ± 0.0 μmol L^−1^ SRP, 9.2 ± 0.2 μmol L^−1^ silicate, and 3.7 ± 1.0 nmol L^−1^ Fe, with a N/P ratio of 12.3 ± 3.8, a Si/N ratio of 0.7 ± 0.2, and a Fe/N ratio of 0.30 ± 0.12 nM/μ M (Figures 3, 4). The range of N/P drawdown ratios for all treatments was 16–21, and the range for Si/N was 0.2–0.4 (Figure 5).

The initial chl *a* concentration was 0.92 ± 0.0 mg m^−3^. All treatments had final concentrations similar to the control (6.1–7.2 mg m^−3^, Figure 6). The phytoplankton population at M2 was dominated by *Pseudonitzschia* spp. Bottles receiving N additions had a lower percentage (39%) of chain-forming pennate diatoms compared to the control and Fe addition treatments (69–71%, Figures 7, 8). Centric diatoms were common at this site across all treatments, and the majority (77–87%) were chain-forming (Figures 7, 8). Picoeukaryotes (range across treatments = 49–57 × 10^3^ cells mL^−1^) outnumbered *Synechococcus* (range across treatments = 6–7 × 10^3^ cells mL^−1^) (Figure 7).

## Discussion

Coastal California nutrient availability creates a mosaic of nutrient limitation patterns that vary with shelf width, distance from shore, and timing and extent of upwelling. In this study we examined five Fe replete sites close to the central California coastline as upwelling relaxed (Figure 2), when N and Fe levels were both elevated (Figures 2, 3). Even within this relatively small geographical area, the waters in this study showed a high degree of spatial heterogeneity with respect to nutrient levels, phytoplankton populations, and biomass nutrient limitation characteristics. Concentrations of nitrate, SRP, silicate, and Fe were approximately twice as high at stations PR and SF than at stations M0, M1, and M2 (Figures 2, 3), likely because they are influenced by the very strong upwelling center at Bodega Bay (Figure 1). Stations PR and SF also had higher ratios of Si:N, although the ratios of N:P were similar among all five sites (Figure 4).

**Figure 9:**
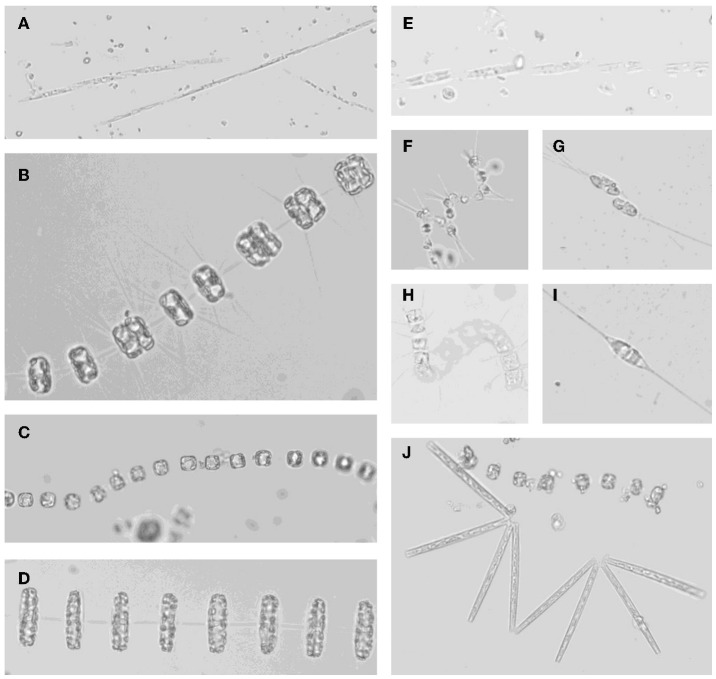
**Micrographs of (A) *Pseudonitzschia* spp.; (B) *Thalassiosira* sp. with dividing cells; (C) *Thalassiosira* sp.; (D) *Thalassiosira rotula*; (E) *Pseudonitzschia* sp.; (F) *Asterionellopsis glacialis*; (G) *Cylindrotheca closterium* dividing cells; (H) unknown chain forming centric diatom; (I) *Cylindrotheca closterium*; (J) *Thalassionema* sp. with *Thalassiosira* sp**.

The plankton community compositions differed considerably among sites. The phytoplankton community near Monterey Bay at stations M0, M1, and M2 was dominated by *Pseudonitzschia* spp. (Figure 9). Picophytoplankton comprised the vast majority of cells in station SF, whereas station PR was populated by *Thalassiosira* spp., and virtually no pennate diatoms were observed (see Supplemental Figures). Grazer populations also varied by location; stations M0, M1, and M2 supported diverse populations of tintinnids (see Supplemental Figure 7), whereas station SF samples contained copepods and copepod fragments, as well as *Protoperidinium* sp. No grazers were observed in samples from station PR. The patchy distribution of populations is consistent with prior observations in Monterey Bay, where phytoplankton species trade off dominance over small spatial scales and time periods (Ryan et al., 2011; Mackey et al., 2012).

Nutrient limitation patterns for biomass also varied by location. All sites had N:P ratios of ~12; this would typically suggest N limitation (because it is lower than the Redfield Ratio of 16:1), however, only three stations (PR, SF, and M1) showed evidence of N limitation based on chl *a* responses in the incubation experiments (Figure 6). At those sites, more N tended to be preferentially drawn down relative to P in bottles receiving additional N (higher N:P drawdown ratio, Figure 5). This suggests that N uptake rates were not saturated even though ambient nitrate levels were high, because added nitrate increased the N uptake rate. This could indicate luxury uptake of N by the diatoms, many of which store nitrate in large vacuoles (Lomas and Gilbert, 2000). In contrast, at stations M0 and M2 all treatments led to similar chl *a* increases, suggesting that nutrient levels were high enough to saturate phytoplankton at these sites and biomass was not nutrient limited.

As expected for the broad shelf regions tested in this study, phytoplankton were not Fe-limited in our experiments. This differs from the nearby California transition zone, where Fe levels are routinely below 1 nM (Biller and Bruland, 2014). The Fe levels at sites in this study ranged from ~4 to 10 nM (Figure 3) comparable to the range of total dissolved Fe reported previously for this part of the California coast (0.3–10 nM; Bruland et al., 2001). Additionally, Fe/N ratios in the transition zone were generally below 0.05 nM/μ M (Biller and Bruland, 2014), which is well below the threshold for Fe limitation in oceanic (0.07) and coastal (0.20) diatoms (Sunda and Huntsman, 1995). Only in the stations closest to shore did Biller and Bruland report Fe/N ratios exceeding the Fe-replete threshold, where values reached up to 0.20, similar to ratios we observed in this study (0.24–0.33; Figures 4C,D). Moreover, natural populations of diatoms can take up excess iron to go through a number of cell divisions when Fe becomes scarce (Yoshida et al., 2006; Sugie et al., 2011), which could further explain the lack of biomass Fe limitation in our bottles.

Changes in the chain forming behavior of diatoms has been linked to Fe and N availability (Hutchins and Bruland, 1998) and temperature (Takabayashi et al., 2006). In this study Fe addition did not cause deviations from the control with respect to chain forming behavior in pennate diatoms (Figure 8). However, Fe additions did appear to enhance chain forming morphology in centric diatoms slightly, though the effect was not significant at *p* < 0.05 (Figure 8). This behavior is consistent with the findings of Hutchins and Bruland (1998), where Fe additions caused more fast sinking, chain forming centric diatoms to bloom. The incubation results suggest that the Fe/N ratios at sites in this study were already high enough (0.24–0.33 nM/μ M) to support chain forming behavior in pennate diatoms in the control and Fe addition samples, thus further Fe additions did not cause any change (Figures 7, 8).

Major differences in the drawdown of Si relative to N were observed among the sites (Figures 5C,D). Stations M0, M1, and M2 all had Si/N drawdown ratios well below the 1:1 Si/N drawdown ratio expected for diatoms (even though these sites were dominated by the diatom *Pseudo-nitzschia* spp.), while stations SF and PR had drawdown ratios close to 1:1. Therefore, less Si than expected was taken up in Monterey Bay compared to the other sites. Interestingly, Si/N drawdown did not show a consistent response to either Fe or N additions (and therefore Fe/N ratios). This response differs from Fe-limited sites along the CA coast where Fe additions caused diatoms to incorporate up to 50% less Si into their frustules relative to N during incubation experiments (Hutchins and Bruland, 1998). The discrepancy in Si/N drawdown among sites suggests factors other than Fe availability influence the drawdown of Si relative to N. One possibility could be differences in Si drawdown between different diatom species, as stations M0, M1 and M2 were dominated by pennate diatoms, whereas SF and PR had more centric diatoms. Alternately, as Si was depleted at M0, M1, and M2, cells may have begun taking up Si and N at different rates, where luxury N uptake could drive lower Si:N drawdown.

Global change presents the possibility for a number of alterations to the biogeochemistry of coastal California (Capone and Hutchins, 2013 and references therein). The flux of nitrate is expected to increase over the next century, and areas that are currently Fe replete could transition toward Fe limitation as the Fe/N ratio decreases (Rykaczewski and Dunne, 2010). In this study, the transition toward higher nitrate flux (lower Fe/N ratios) was simulated by nitrate additions in the incubation experiment. These additions of 10 μmol/L nitrate are within the range of predicted future N increases, and brought Fe/N ratios into Fe-limited range (range of Fe/N = 0.16–0.18 nmol/μ mol) at each site except for station PR (Fe/N = 0.25 nmol/ μ mol).

**Figure 10:**
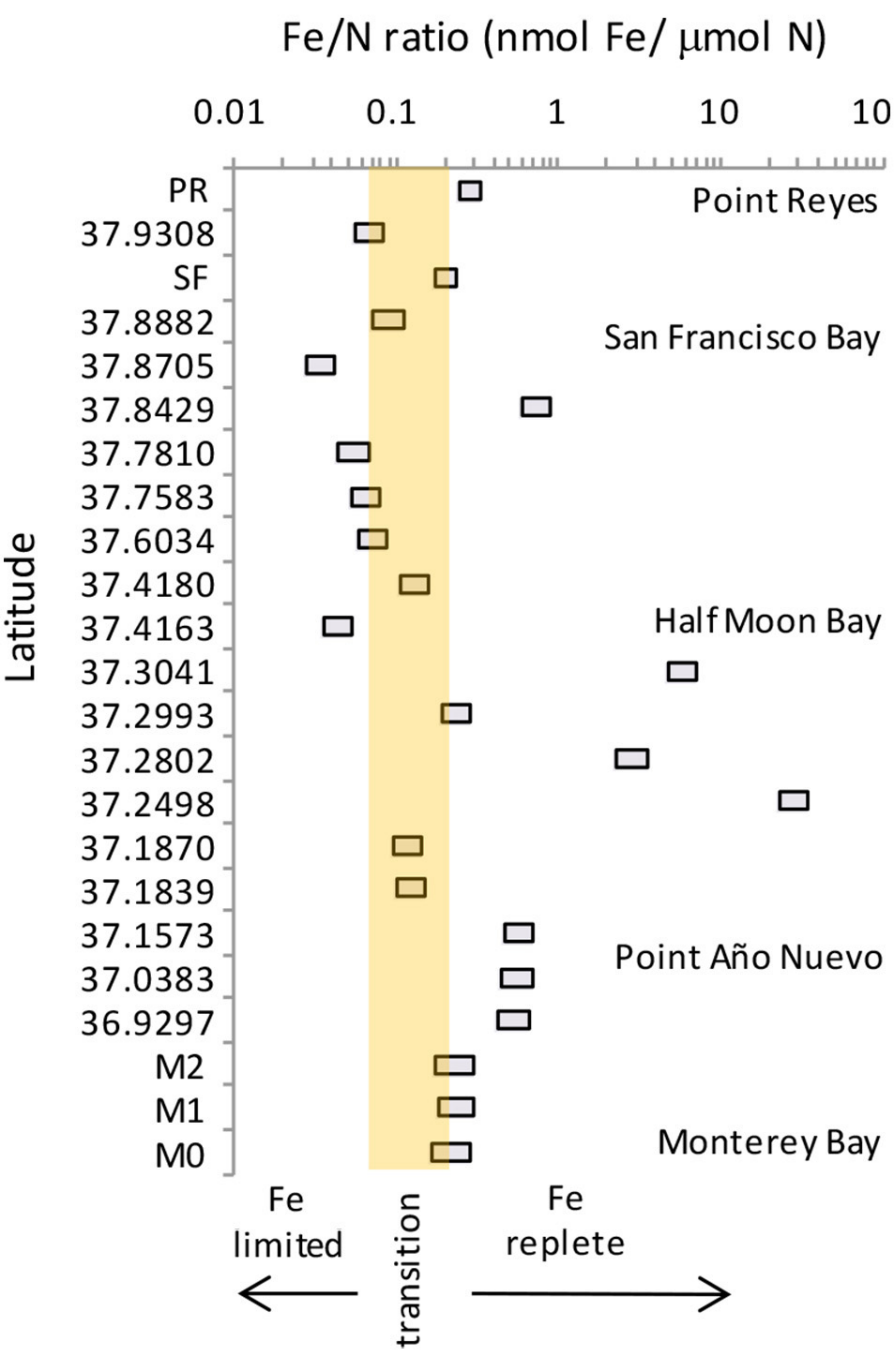
**Range of Fe/N ratios for the region between Monterey Bay and Point Reyes**. Boxes show the present day (right hand side of box) and projected future Fe/N ratio (left hand side of box). Shaded region denoted the range of Fe/N ratios (0.07–0.2) over which oceanic and coastal diatoms become Fe-limited. Data are from this study for stations PR, SF, M0, M1, and M2, and from table 1 of Biller et al. (2013) for all other latitudes. Projected future ratios were calculated assuming a 50% increase in nitrate as predicted by Rykaczewski and Dunne (2010).

We compared our data to published values of Fe and N for the region spanning from Monterey Bay to Point Reyes (Biller et al., 2013), and calculated the potential shift in Fe/N ratio assuming a 50% increase in nitrate at each site as projected by Rykaczewski and Dunne (2010) (Figure 10). While biomass at many of the locations in this region are currently Fe replete, others are closer to becoming Fe-limited. Coastal diatoms become Fe-limited below a Fe/N ratio of 0.20 nmol/μ mol, and oceanic species have a slightly lower threshold ratio (0.07 nmol/μ mol; Sunda and Huntsman, 1995). Thirteen of the 23 sites we compared would be considered Fe replete under present day conditions (>0.2 nmol Fe/μ mol N); however, biomass at five of these locations (e.g., within Monterey Bay, south of Half Moon Bay, and along the coast near San Francisco Bay) would cross into the Fe-limited range in the future if nitrate flux increases. The ratios of the other 10 sites that are presently Fe-limited would of course also decline further. These shifts could lead to succession of phytoplankton species better adapted to dealing with Fe limitation, as well as changes in chain forming behavior as observed in this study.

Increased nitrate flux is also anticipated to cause a concomitant increase in productivity in coastal California (Rykaczewski and Dunne, 2010). Addition of nitrate increased phytoplankton biomass in three of the five sites assayed in this study, leading to a 28–60% increase in chl *a* (Figure 6) and increasing the drawdown of N relative to P (Figures 5A,B). Nitrogen addition also affected the chain-forming behavior of phytoplankton by encouraging the growth of slower sinking, single-celled pennate diatoms in all of the sites (Figure 8A). This was due to both a physiological shift in *Pseudonitzschia* spp. toward a single-celled growth habit, as well as a community shift toward species like *Cylindrotheca* spp. that naturally tend to exist as solitary cells.

These changes in phytoplankton physiology and community structure suggest that enhanced N flux could lead to ecological and biogeochemical shifts in the California upwelling system in the future. First, the sinking rate of cells, and hence the export of C, Si, and other elements could decline with the shift to smaller chains of cells with slower sinking rates. Second, these shifts have the potential to propagate up the food web because different, potentially smaller grazers could be favored by the increase in single cells. And finally, if increased N flux favors pennate species other than *Pseudonitzschia* spp. as our experiments suggest, then blooms of this potentially toxin-producing genus could become less prevalent in the future. Indeed, the toxin producing species *Pseudonitszchia pungens* is more competitive at low N:P ratios of ~10:1 compared to more N rich waters (Hu et al., 2008). The N:P ratios in our unamended water ranged from 12.3 to 13.4, whereas the nitrate-treated water ranged from 18.0 to 21.8. It is therefore not surprising that the slightly N limited conditions and low N/P ratios in the present-day water would favor *Pseudonitszchia*, which leaves open the possibility that *Pseudonitszchia* could become less competitive due to excess N in the future.

Changes in ecosystem services, which are the benefits people derive from marine ecosystems, could also be affected by the changes described here. In California, a major ecosystem service provided by coastal waters is fishery yield. Many studies have shown a link between the amount of upwelling that occurs in a given year and the production of fisheries (Gunsolus, 1978; Nickelson, 1986). The relationship is also apparent in comparing fishery production along the west coast of North and South America during El Nino (low upwelling) and La Nina (high upwelling) years. If future primary productivity increases affect fisheries in a similar manner to natural increases in upwelling, it is possible that CA fisheries could become more productive in the future. The phytoplankton population shift toward smaller cells, which decreases export production due to a decrease in sinking rates, could likewise increase fisheries yields by providing more carbon biomass to grazers.

The projected increase in N flux by the year 2100 is expected to coincide with an 18% decrease in oxygen concentration (Rykaczewski and Dunne, 2010) and a decrease in pH by 0.5 units due to anthropogenic ocean acidification (Doney et al., 2009). These multiple stressors could exacerbate or mitigate the effects observed in this study. In particular, the effect of changing seawater chemistry on Fe solubility is difficult to predict. Ocean acidification has the potential to reduce Fe bioavailability by protonating Fe ligands, causing them to retain Fe ions (Shi et al., 2010). In contrast, expansion and shoaling of hypoxic zones would serve to increase Fe solubility, because the reduced Fe(II) in hypoxic waters is more soluble than the Fe(III) in oxygenated waters. In this study Fe additions caused little change in the phytoplankton community because cells were already Fe replete; however, future shifts toward Fe limitation could enhance the importance of soluble Fe supply in these regions (Figure 10). Moreover, large blooms of phytoplankton increase the pH of seawater, which could partially or wholly offset the local effects of ocean acidification on Fe availability. It is therefore not clear whether Fe availability will increase or decrease in the future, or what effect, if any, this will have on marine biota in upwelling regions. The Canary and Benguela upwelling systems, which are more Fe replete than the California and Peru systems, are likely to be less sensitive to these changes in Fe biogeochemistry, and changes in N flux may be more important.

Upwelling systems are naturally variable regions inhabited by organisms that thrive under changing conditions. In regions like coastal California where phytoplankton biomass is already on the cusp between N and Fe limitation, modest shifts in nutrient supply ratios could elicit important changes in cell physiology, community composition, and nutrient uptake. This study shows that increased N availability in recently upwelled water leads to faster cell growth, greater nitrate drawdown, and favors solitary cells over chain-formers. Upwelling centers throughout the world's oceans are morphologically and biogeochemically diverse, and future studies should help determine if similar responses could occur in other upwelling regions.

### Conflict of interest statement

The authors declare that the research was conducted in the absence of any commercial or financial relationships that could be construed as a potential conflict of interest.
