# Supplementary material for: Microbial and biogeochemical responses to projected future nitrate enrichment in the California upwelling system
Source: Front Microbiol. 2014 Nov 20;5:632. doi: 10.3389/fmicb.2014.00632 (PMC4238378; doi:10.3389/fmicb.2014.00632)
Supplement: Supplementary file 1 [file DataSheet1.DOCX]

Supplemental table 1:

| Detection limit and CRM analyses. Units are nmol/kg if not mentioned. | | | | | | | |  | |  |
| --- | --- | --- | --- | --- | --- | --- | --- | --- | --- | --- |
| Metal | Detection limit* |  | SAFe_S (n=6)** | | | | | |  |  |
|  |  |  | measured | consensus | | | | |  |  |
|  |  |  | av ± SD | | | | | |  |  |
| Al | 2.28 |  | BD | 1.67 ± 0.10 | | | | |  |  |
| Mn | 0.023 |  | 0.84 ± 0.05 | 0.79 ± 0.06 | | | | |  |  |
| Fe | 0.74 |  | BD | 0.093 ± 0.008 | | | | |  |  |
| Co | 0.005 |  | 0.003 ± 0.002 | 0.005 ± 0.001 | | | | |  |  |
| Ni | 0.11 |  | 2.45 ± 0.17 | 2.28 ± 0.09 | | | | |  |  |
| Cu | 0.013 |  | 0.37 ± 0.02 | 0.52 ± 0.05 | | | | |  |  |
| Zn | 0.24 |  | BD | 0.069 ± 0.010 | | | | |  |  |
| Cd | 0.0004 |  | 0.0017 ± 0.0008 | 0.0011 ± 0.0003 | | | | |  |  |
| Pb | 0.0009 |  | 0.044 ± 0.001 | 0.0480 ± 0.0022 | | | | |  |  |
| * Detection limit calculated by 3×standard deviation of the blank. | | | | |  | | |  | |  |
| **no UV treatments | | | | |  |  |  | |  |  |

Supplemental Table 1: Mean ± standard error cell concentration data; n=3 replicates for each treatment at each site. Concentration units are in (cells mL^-1^).

| Station | Treatment | Single-cell pennate diatoms | Chain-forming pennate diatoms | % Chain-forming pennate diatoms | Single-cell centric diatoms | Chain-forming centric diatoms | % Chain-forming centric diatoms | Other | Picoeukaryotes | *Synechococcus* |
| --- | --- | --- | --- | --- | --- | --- | --- | --- | --- | --- |
| PR | Control | 30 ± 4 | 21 ± 7 | 40 ± 8 | 112 ± 11 | 499 ± 128 | 81 ± 2 | 8 ± 7 | 78294 ± 10706 | 4781 ± 499 |
| PR | Fe | 30 ± 7 | 32 ± 8 | 51 ± 7 | 98 ± 12 | 439 ± 68 | 82 ± 1 | 4 ± 2 | 89404 ± 6615 | 5022 ± 212 |
| PR | N | 32 ± 6 | 30 ± 8 | 47 ± 7 | 103 ± 23 | 298 ± 100 | 71 ± 8 | 4 ± 3 | 96652 ± 5787 | 7053 ± 1619 |
| SF | Control | 4 ± 1 | 0 ± 0 | 6 ± 1 | 34 ± 1 | 16 ± 6 | 30 ± 7 | 3 ± 1 | 197639 ± 37446 | 9367 ± 1312 |
| SF | Fe | 5 ± 1 | 0 ± 0 | 6 ± 4 | 39 ± 7 | 29 ± 7 | 41 ± 3 | 2 ± 0 | 264084 ± 20738 | 8916 ± 1855 |
| SF | N | 5 ± 1 | 0 ± 0 | 0 ± 0 | 46 ± 9 | 13 ± 1 | 23 ± 4 | 2 ± 0 | 276031 ± 8551 | 7547 ± 224 |
| M0 | Control | 5 ± 0 | 25 ± 3 | 82 ± 2 | 11 ± 4 | 8 ± 1 | 46 ± 9 | 7 ± 3 | 53841 ± 2624 | 6087 ± 347 |
| M0 | Fe | 8 ± 1 | 28 ± 4 | 78 ± 2 | 13 ± 2 | 18 ± 4 | 57 ± 2 | 6 ± 2 | 56271 ± 6157 | 7375 ± 504 |
| M0 | N | 14 ± 4 | 20 ± 2 | 60 ± 6 | 19 ± 3 | 10 ± 1 | 34 ± 2 | 6 ± 2 | 56654 ± 6790 | 6100 ± 198 |
| M1 | Control | 13 ± 1 | 29 ± 3 | 70 ± 3 | 6 ± 1 | 15 ± 5 | 67 ± 10 | 13 ± 2 | 63766 ± 4873 | 9135 ± 288 |
| M1 | Fe | 28 ± 5 | 49 ± 4 | 64 ± 6 | 8 ± 2 | 15 ± 4 | 64 ± 5 | 16 ± 1 | 65479 ± 4092 | 7106 ± 640 |
| M1 | N | 25 ± 4 | 34 ± 9 | 56 ± 8 | 6 ± 1 | 21 ± 7 | 72 ± 8 | 14 ± 2 | 64716 ± 3577 | 8562 ± 545 |
| M2 | Control | 16 ± 5 | 40 ± 7 | 71 ± 7 | 6 ± 0 | 22 ± 6 | 77 ± 6 | 2 ± 1 | 25341 ± 1224 | 3220 ± 91 |
| M2 | Fe | 22 ± 7 | 40 ± 10 | 65 ± 5 | 6 ± 0 | 33 ± 7 | 84 ± 3 | 1 ± 1 | 27302 ± 4312 | 3391 ± 48 |
| M2 | N | 44 ± 3 | 28 ± 2 | 39 ± 3 | 6 ± 1 | 46 ± 13 | 87 ± 3 | 1 ± 0 | 24485 ± 3145 | 2962 ± 688 |
