# Supplementary figures and images for: Microbial and biogeochemical responses to projected future nitrate enrichment in the California upwelling system
Source: Front Microbiol. 2014 Nov 20;5:632. doi: 10.3389/fmicb.2014.00632 (PMC4238378; doi:10.3389/fmicb.2014.00632)

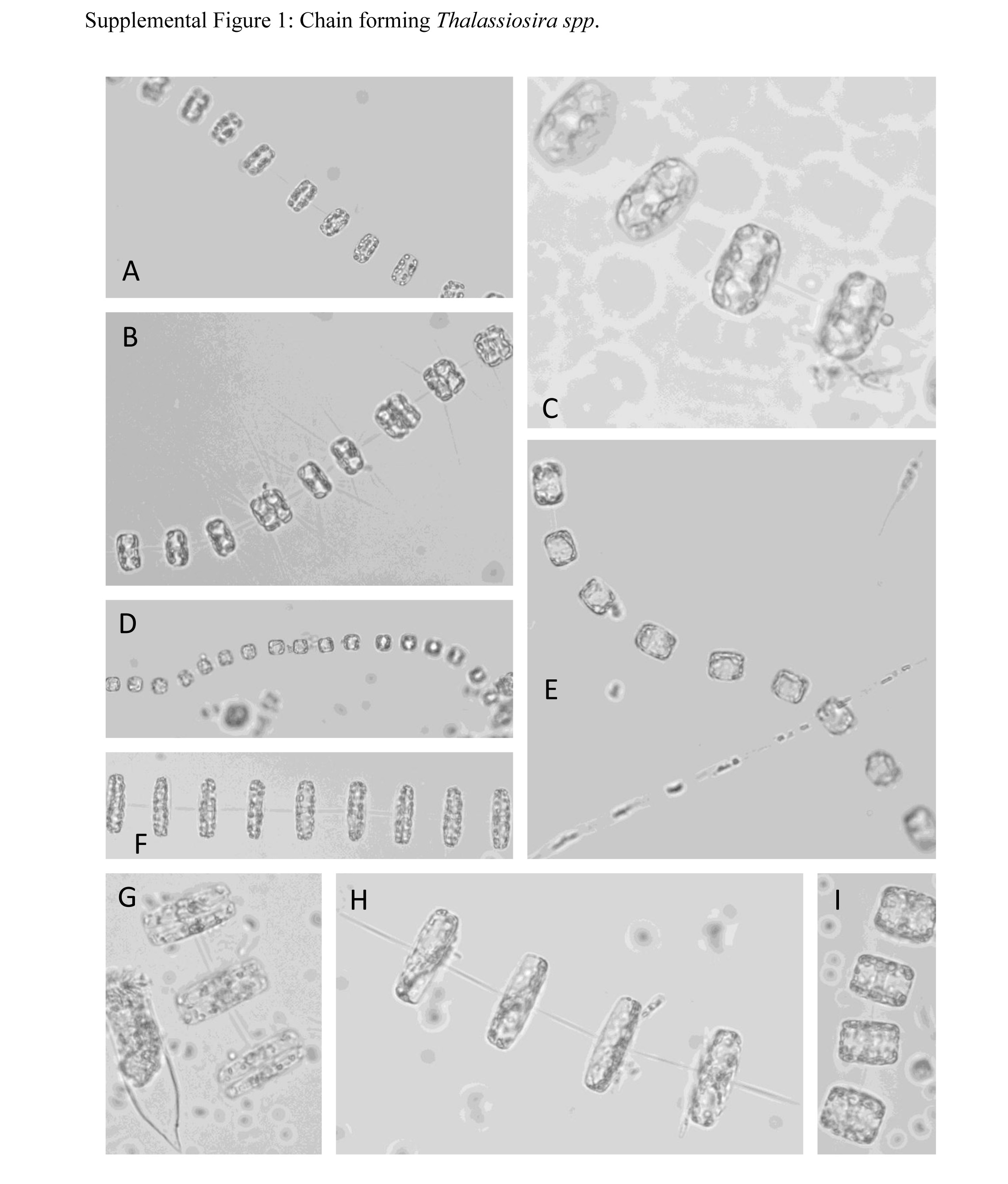

Supplement: Supplementary file 2 [file DataSheet2.ZIP › Supplemental Figures/S1.jpg]

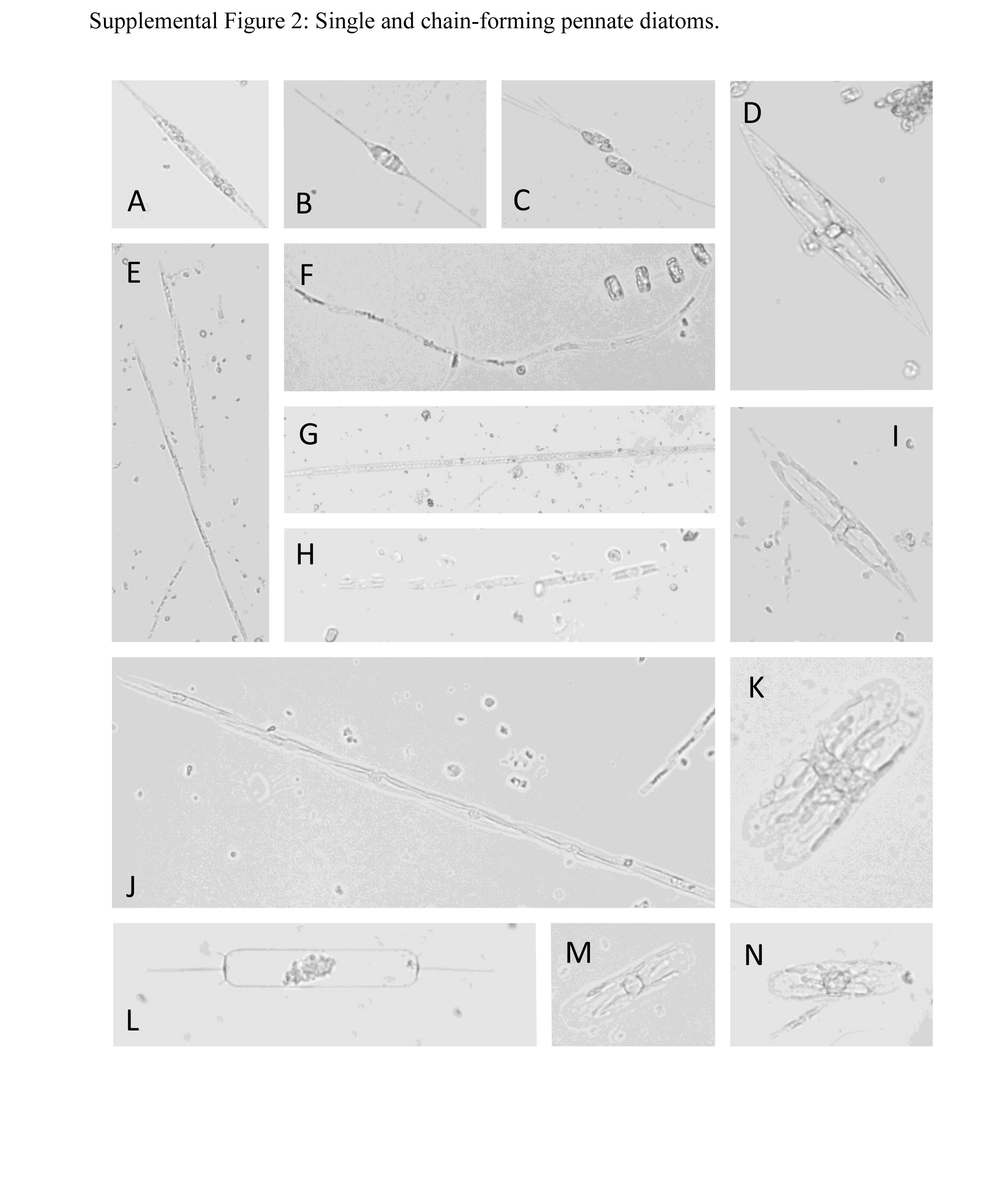

Supplement: Supplementary file 2 [file DataSheet2.ZIP › Supplemental Figures/S2.jpg]

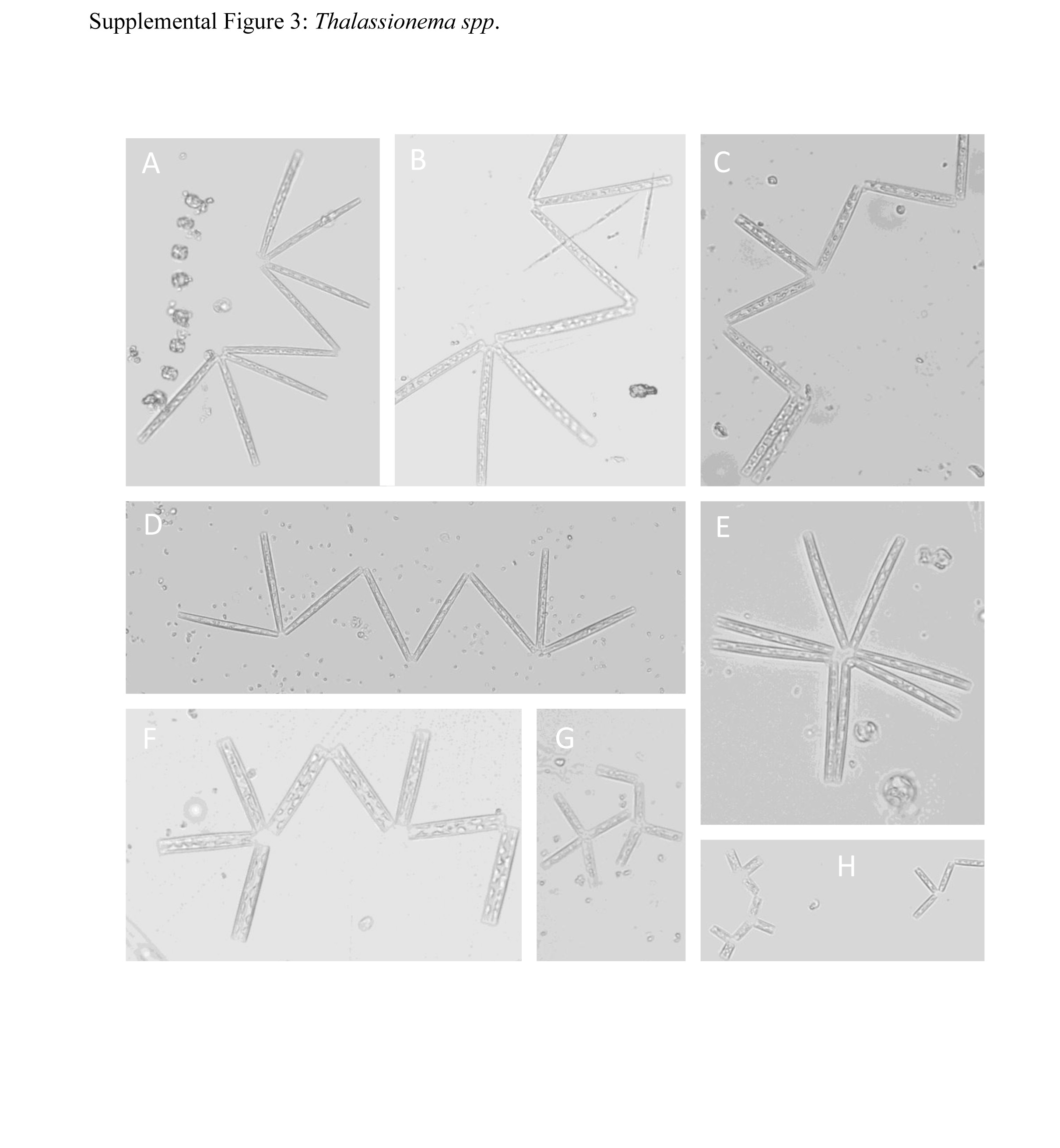

Supplement: Supplementary file 2 [file DataSheet2.ZIP › Supplemental Figures/S3.jpg]

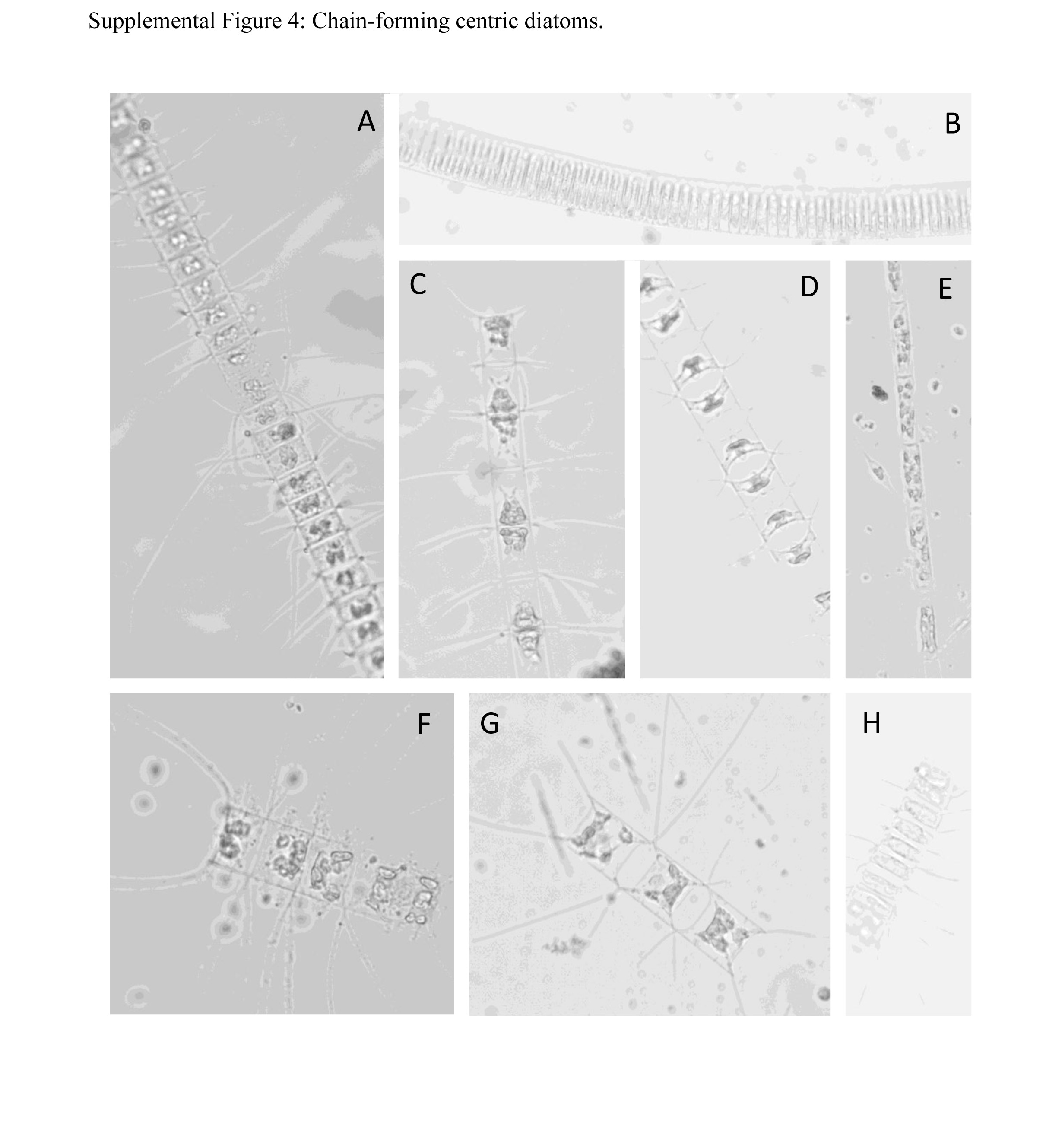

Supplement: Supplementary file 2 [file DataSheet2.ZIP › Supplemental Figures/S4.jpg]

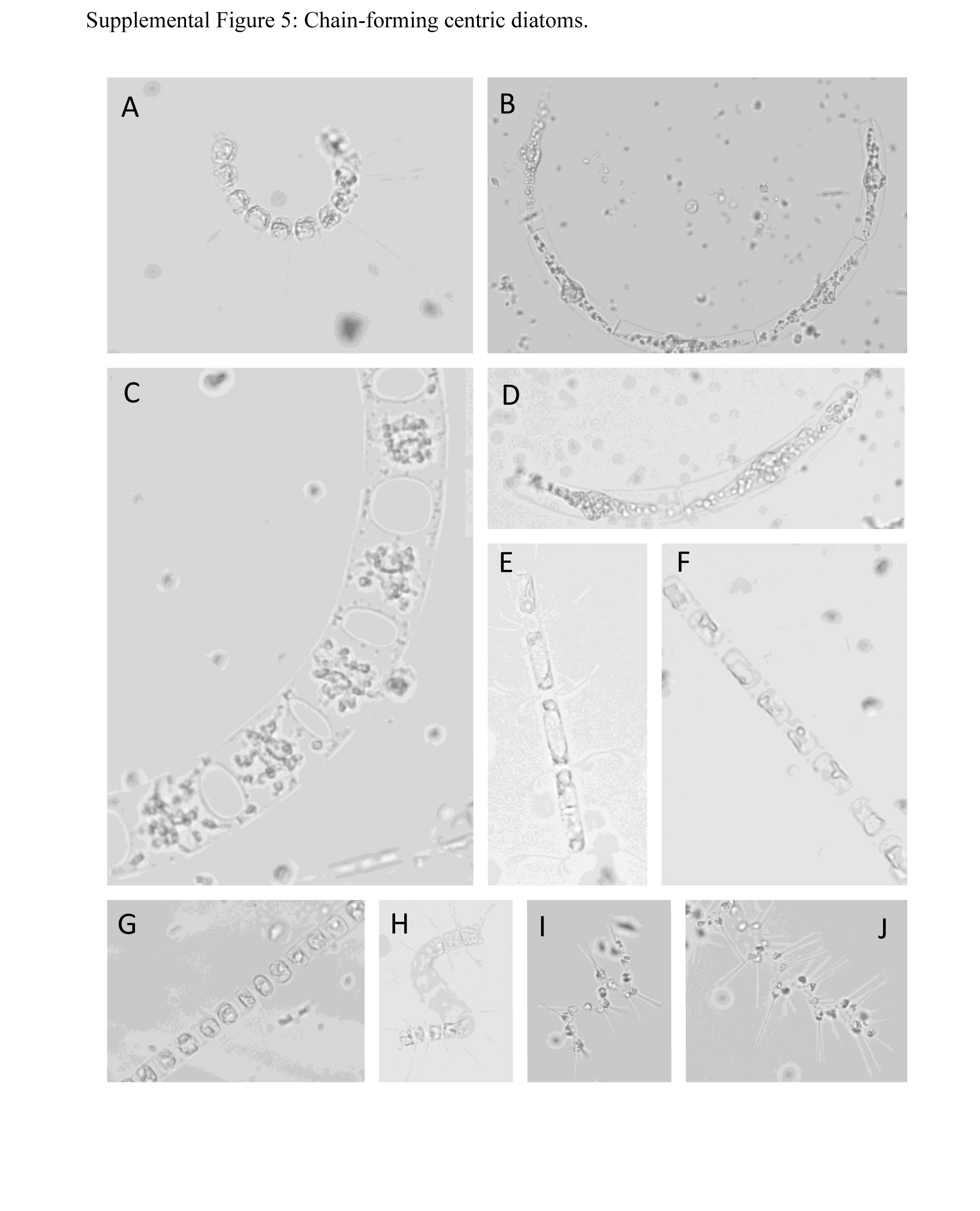

Supplement: Supplementary file 2 [file DataSheet2.ZIP › Supplemental Figures/S5.jpg]

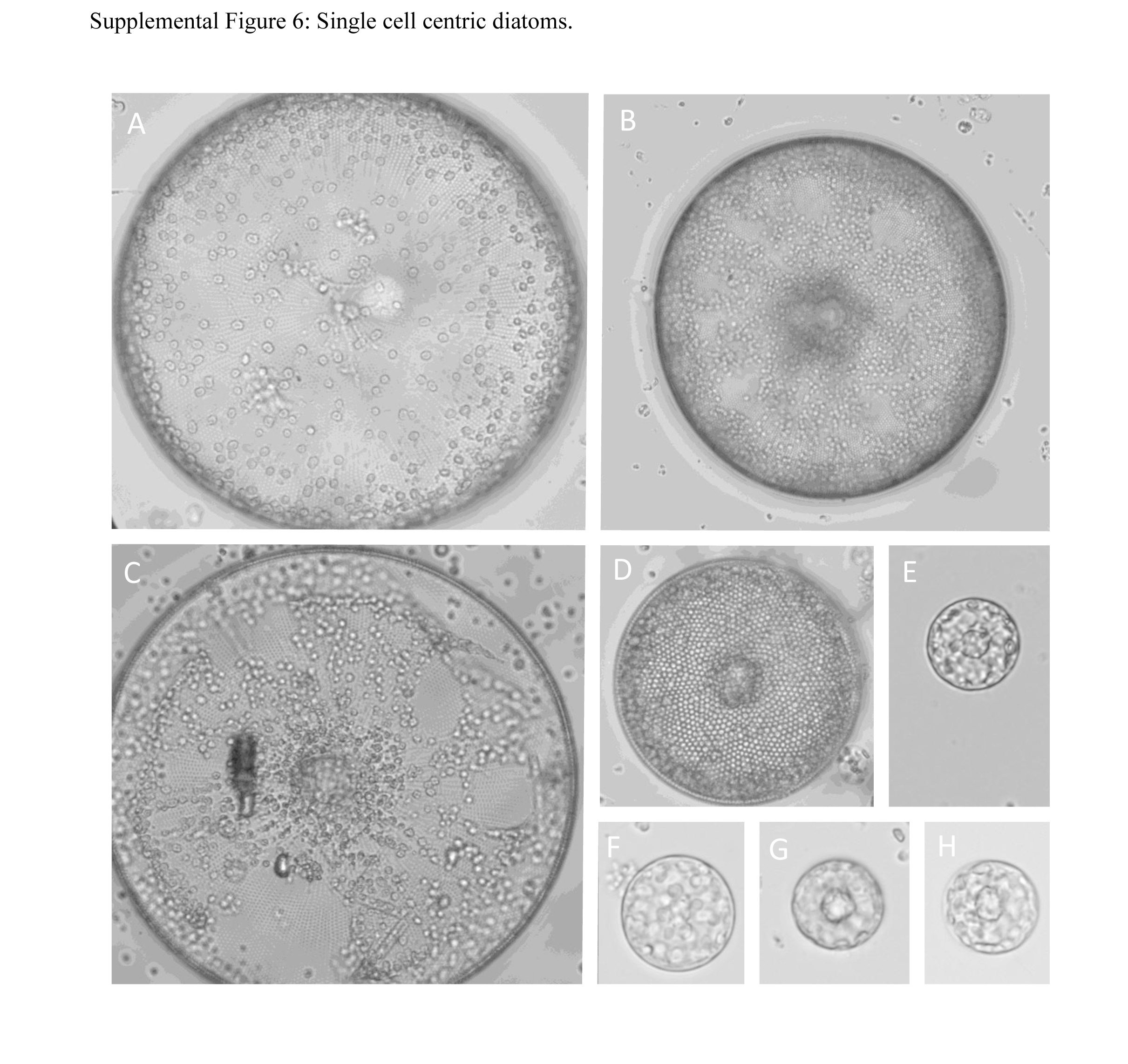

Supplement: Supplementary file 2 [file DataSheet2.ZIP › Supplemental Figures/S6.jpg]

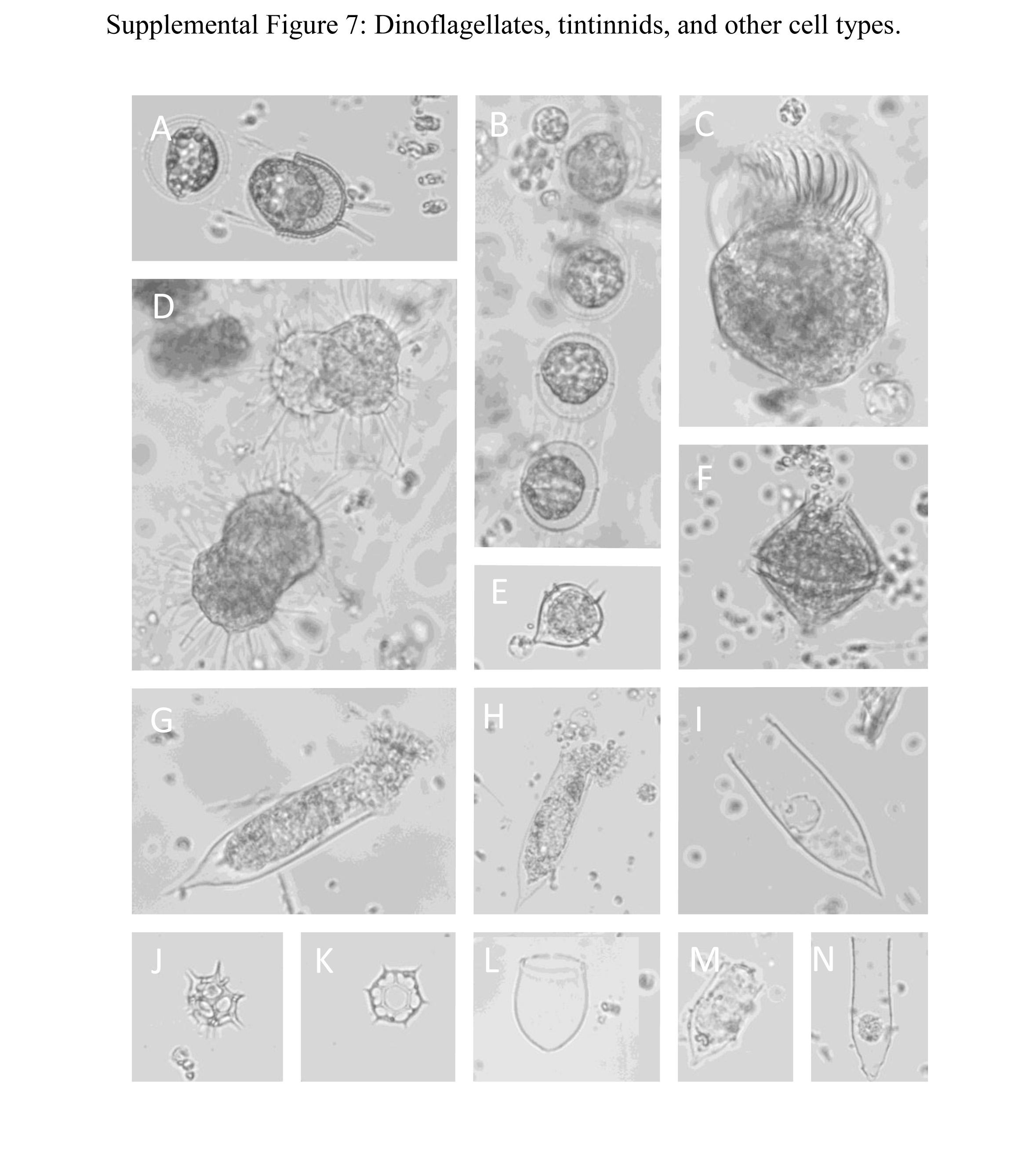

Supplement: Supplementary file 2 [file DataSheet2.ZIP › Supplemental Figures/S7.jpg]

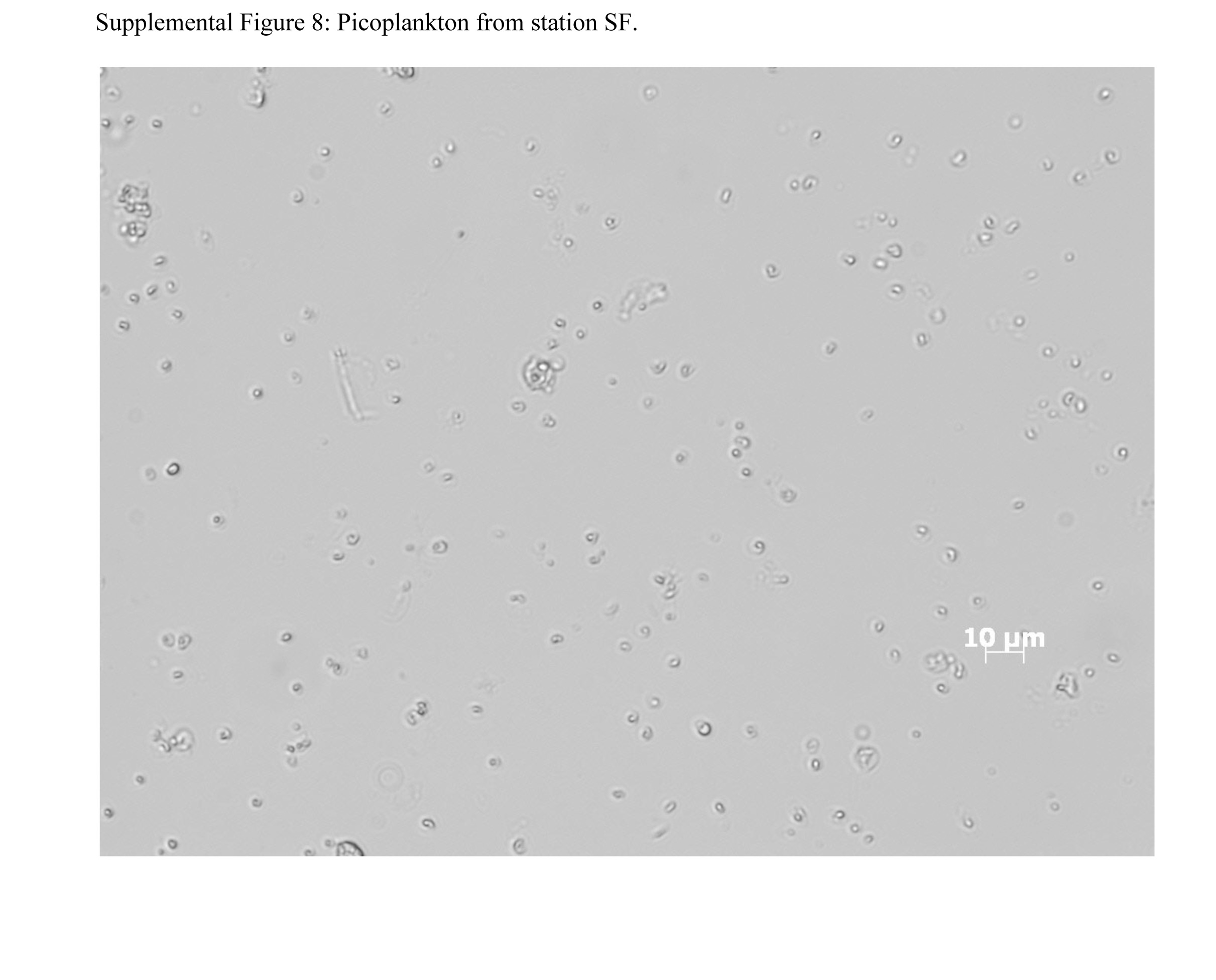

Supplement: Supplementary file 2 [file DataSheet2.ZIP › Supplemental Figures/S8.jpg]

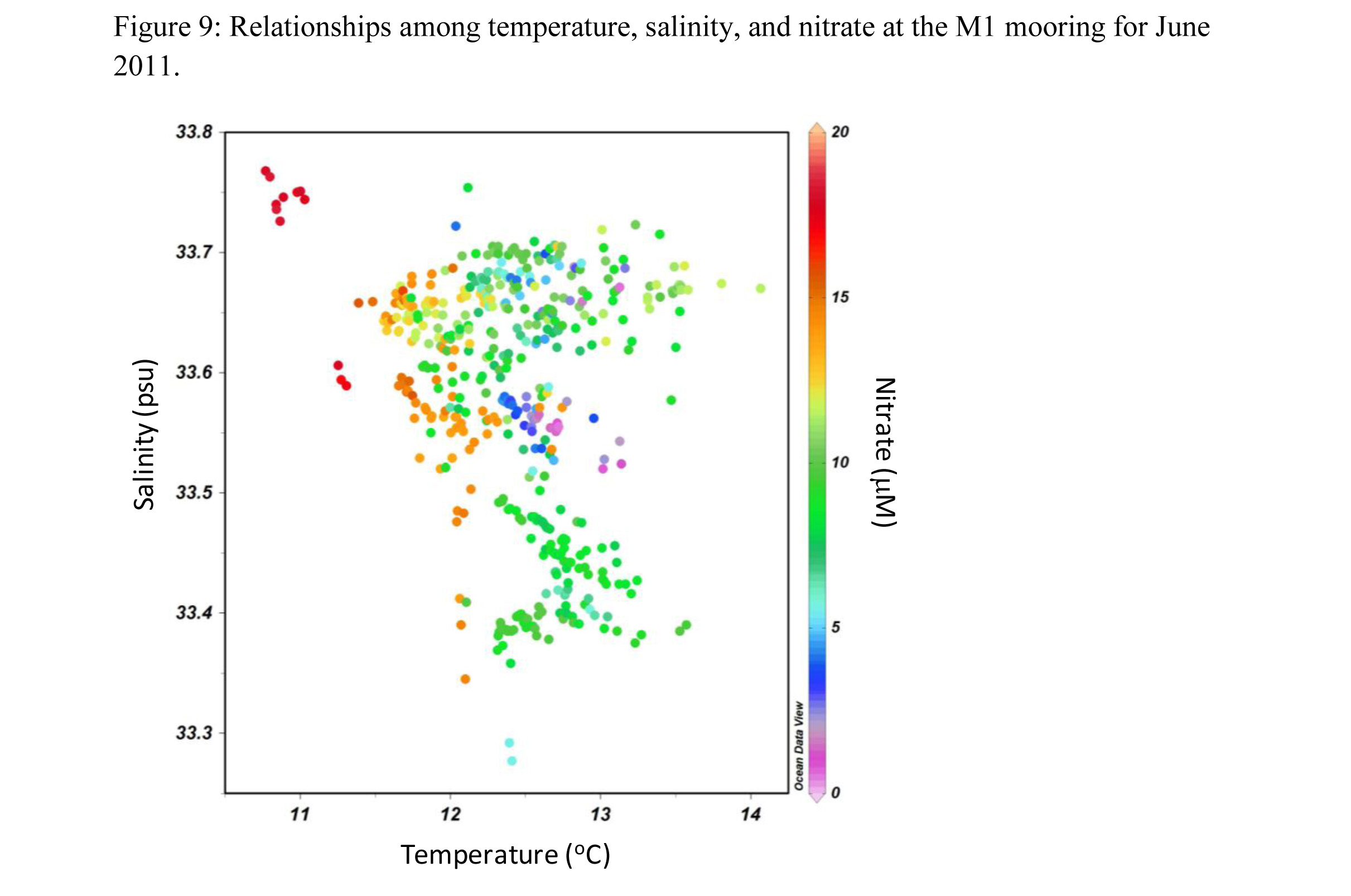

Supplement: Supplementary file 2 [file DataSheet2.ZIP › Supplemental Figures/S9.jpg]
